# What affected UK adults’ adherence to medicines during the COVID-19 pandemic? Cross-sectional survey in a representative sample of people with long-term conditions

**DOI:** 10.1007/s10389-022-01813-0

**Published:** 2023-01-19

**Authors:** L. S. Penner, C. J. Armitage, T. Thornley, P. Whelan, A. Chuter, T. Allen, R. A. Elliott

**Affiliations:** 1 Janssen-Cilag GmbH, Johnson & Johnson Platz 1, 41470 Neuss, Germany; 2 Manchester Centre for Health Economics, Division of Population Health, Health Services Research and Primary Care, School of Health Sciences, Faculty of Biology, Medicine and Health, University of Manchester, 4th Floor, Jean McFarlane Building, Manchester, M13 9PL UK; 3 Manchester Centre for Health Psychology, Division of Psychology and Mental Health, School of Health Sciences, Faculty of Biology, Medicine and Health, University of Manchester, Coupland Building 1, Manchester, M13 9PL; Manchester University NHS Foundation Trust, Manchester Academic Health Sciences Centre, Manchester, M13 9PT; NIHR Greater Manchester Patient Safety Translational Research Centre, University of Manchester, Coupland Building 1, Manchester, M13 9PL UK; 4 School of Pharmacy, University of Nottingham, University Park, Nottingham, NG7 2RD UK; 5 Digital Health Technical Lead, Centre for Health Informatics, Division of Informatics, Imaging and Data Science| School of Health Sciences | Faculty of Biology, Medicine and Health, University of Manchester, Vaughan House, Manchester, M13 9PL UK; 6 Danish Centre for Health Economics, University of Southern Denmark, Odense, Denmark

**Keywords:** Behaviour and behaviour mechanisms, Community pharmacy services, COVID-19, Primary healthcare, Treatment adherence and compliance

## Abstract

**Aim:**

Medicines non-adherence is associated with poorer outcomes and higher costs. COVID-19 affected access to healthcare, with increased reliance on remote methods, including medicines supply. This study aimed to identify what affected people’s adherence to medicines for long-term conditions (LTCs) during the pandemic.

**Subject and methods:**

Cross-sectional online survey of UK adults prescribed medicines for LTCs assessing self-reported medicines adherence, reasons for non-adherence (using the capability, opportunity and motivation model of behaviour [COM-B]), medicines access and COVID-19-related behaviours.

**Results:**

The 1746 respondents reported a mean (SD) of 2.5 (1.9) LTCs, for which they were taking 2.4 (1.9) prescribed medicines, 525 (30.1%) reported using digital tools to support ordering or taking medicines and 22.6% reported medicines non-adherence. No access to at least one medicine was reported by 182 (10.4%) respondents; 1048 (60.0%) reported taking at least one non-prescription medicine as a substitute; 409 (23.4%) requested emergency supply from pharmacy for at least one medicine. Problems accessing medicines, being younger, male, in the highest socioeconomic group and working were linked to poorer adherence. Access problems were mostly directly or indirectly related to the COVID-19 pandemic. Respondents were generally lacking in capabilities and opportunities, but disruptions to habits (automatic motivation) was the major reason for non-adherence.

**Conclusion:**

Navigating changes in how medicines were accessed, and disruption of habits during the COVID-19 pandemic, was associated with suboptimal adherence. People were resourceful in overcoming barriers to access. Solutions to support medicines-taking need to take account of the multiple ways that medicines are prescribed and supplied remotely.

**Supplementary Information:**

The online version contains supplementary material available at 10.1007/s10389-022-01813-0.

## Introduction

Worldwide, about 25% of medicines prescribed for long-term conditions (LTCs) (DiMatteo 2004) are not taken as directed, causing avoidable morbidity, mortality, healthcare and societal costs (Ho et al. 2006; Vestbo et al. 2009; Trueman et al. 2010; Chowdhury et al. 2013; Cutler et al. 2018; Mongkhon et al. 2018). The introduction of COVID-19-related measures in the United Kingdom (UK) in March 2020 meant that physical distancing, lockdown regulations and COVID-19-avoidance behaviours were likely to have exacerbated already suboptimal adherence (Keyworth et al. 2021). Patients with LTCs had less contact with healthcare professionals (Rathbone et al. 2021), fewer face-to-face general practitioner (GP) consultations (Royal College of General Practitioners 2020a, b), increased no-shows for face-to-face consultations (Hong et al. 2021), increasing use of electronic prescribing, repeat dispensing, home delivery services, and digital interventions supporting electronic triage and self-management (Pharmacy Services Negotiating Committee 2020).

In this survey, we asked a representative sample of community-dwelling adults taking regularly prescribed medicines for LTCs about their medicines use, exploring the impact of sociodemographic, medicines-, disease- and system-related factors on medicines use (Horne et al. 2005; Gast and Mathes 2019). We explored the potential effects of the COVID-19 pandemic on medicines use, including access issues and use of digital or remote technology to support medicines use.

## Materials and methods

### Study design

This was a cross-sectional study. Kantar, a market research company, recruited patients and collected responses to requested questionnaire items as part of their twice-weekly “omnibus” online survey designed to assess public opinions. The survey was targeted at people taking prescribed medicines for LTCs. Kantar recruited a sample of UK residents aged 16+ from their existing database in three survey waves representative of the UK population with regards to gender, age, socioeconomic group and household size. The relevant cohort within this sample included only respondents reporting taking at least one medicine for an LTC. Ethical approval was obtained from the University of Manchester Ethics Committee (ref: 2021-11485-19655, 22/06/2021).

The survey collected data on (1) sociodemographic information, (2) LTCs, medicines taken for LTCs, self-reported medicines adherence and reasons for non-adherence, (3) access to medicines (use and barriers to use of GPs, pharmacies, social networks, and digital and remote interventions in obtaining and taking medicines), and (4) COVID-19-related behaviours and experiences. See Appendix 1 for study design and methodology detail and Appendix 2 for the questionnaire.

### Medicines

Respondents reported existing and new medicines they were taking for each LTC. Self-reported non-adherence to medicines was defined as missing at least one dose of a medicine in the last 7 days. The question was introduced with a statement that normalises non-adherence (“People often miss taking their medicines, for a whole range of reasons”), and used a specified and short recall period for adherence behaviour as recommended for adherence measures (Stirratt et al. 2015). This method for detecting non-adherence has been widely used (Clifford et al. 2006; Elliott et al. 2020; Persaud et al. 2021) and was found to report similar results to the Morisky Medication Adherence Scale (MMAS) (Elliott et al. 2020).

We used the capability, opportunity and motivation model of behaviour change (COM-B) (Keyworth et al. 2020), a validated theoretical framework endorsed by the UK National Institute for Health and Care Excellence (National Institute for Health and Care Excellence 2014) to understand reasons for non-adherence. Respondents were asked to report by how much they agreed (strongly disagree [0] to strongly agree [10]) with each of the six items describing potential reasons for non-adherence: physical capability, psychological capability, physical opportunity, social opportunity, reflective motivation, and automatic motivation (Keyworth et al. 2020) [Appendix 1].

### Access to medicines

Respondents were asked about access to GP or community pharmacy services: how prescriptions and medicines were accessed, times with no access to medicine, reasons for access problems, and actions taken to resolve access problems. The questionnaire covered general factors affecting medicines access (prescription fees, prepayment certificates, car ownership, availability of public transport, help with taking medicines at home, and uptake of digital tools to support medicines management) and COVID-19-related factors (shielding status, COVID-19 infection, treatment, and avoidance behaviour).

### Statistical analysis

Descriptive statistics were used to characterise the population, medicines, medicines adherence and factors affecting access. Adherence was expressed as binary variable. Logistic regression analyses were used to model associations between these factors and medicines adherence [see Appendix 1].

## Results

### Response rate

Data were collected via three survey waves on 29th June (*n*=1278), 6th July (*n*=1283) and 8th July 2021 (*n*=1264). This period was immediately prior to the removal of COVID-19 restrictions on 19th July 2021, and no major shifts in government policy occurred between the three survey dates. At the time of the survey, legal limits in place included 2-metres physical distancing rules, wearing face coverings in specific settings and limits on how many people could attend meetings or events (6 people or 2 households indoors, 30 people outdoors) (Cabinet Office 2021). Of the 3825 respondents across the three waves, 1746 (45.6%) reported taking a prescribed medicine for an LTC, and this formed the cohort for our study.

Table 1 provides a summary of key sociodemographic characteristics, factors related to access to medicine, LTCs, medicines and COVID-19-related factors.

**Table 1:** Socio-demographic characteristics of respondents reporting taking at least one medicine for a long-term condition

		Total *N*=1746
Mean age (SD, range)		51.1 (17.2, 16 to 92)
Female		949 (54.4%)
Ethnicity		
White		1529 (87.6%)
Black, Asian and minority ethnic populations		148 (8.5%)
Not responded		69 (4.0%)
Rural or urban area		
Rural		317 (18.2%)
Urban		1236 (70.8%)
Not responded ^**a**^		193 (11.1%)
Working		863 (49.4%)
Household size		
1 person		388 (22.2%)
2 persons		687 (39.3%)
3+ with children		428 (24.5%)
3+ without children		243 (13.9%)
Socioeconomic group		
1 Semi or unskilled manual worker		203 (11.6%)
2 Skilled manual worker		248 (14.2%)
3 Supervisory/Junior managerial/Professional/Administrator		453 (25.9%)
4 Intermediate managerial/Professional/Administrative		395 (22.6%)
5 Higher managerial/Professional/Administrative		110 (6.3%)
Student		26 (1.5%)
Retired and living on state pension		120 (6.9%)
Unemployed		191 (10.9%)
Index of multiple deprivation (IMD)		
Q1 most deprived		530 (30.4%)
Q2		294 (16.8%)
Q3		251 (14.4%)
Q4		250 (14.3%)
Q5 least deprived		230 (13.2%)
Not responded ^**a**^		191 (10.9%)
Car ownership		1305 (74.7%)
Suitable public transport available		1295 (74.2%)
Help taking medicines at home		273 (15.6%)
Exempt from prescription fee		
No/Yes/Unknown ^**b**^		467 (26.7%)/980 (56.1%)/299 (17.1%)
Prepayment certificate for prescriptions		
No/Yes/Unknown		311 (17.8%)/156 (8.9%)/1279 (73.3%)
Mean number of LTCs (SD, range)		2.5 (1.9, 1 to 18)
At least one LTC newly diagnosed		467 (26.7%)
Mean number of LTCs newly diagnosed (SD, range)		0.4 (0.9, 0 to 11)
GI condition		135 (7.7%)
CV condition		581 (33.3%)
Respiratory condition		362 (20.7%)
CNS condition		942 (54.0%)
Mental health condition		585 (33.5%)
Fibromyalgia/chronic widespread pain		67 (3.8%)
Back pain		247 (14.1%)
Migraine		185 (10.6%)
Chronic pain		160 (9.2%)
Epilepsy		45 (2.6%)
Multiple sclerosis		5 (0.3%)
Infectious condition		13 (0.7%)
Endocrine condition		414 (23.7%)
Type 1 diabetes		42 (2.4%)
Type 2 diabetes		213 (12.2%)
Other endocrine condition		183 (10.5%)
Malignant disease		29 (1.7%)
Rheumatic condition		251 (14.4%)
Ophthalmic condition		35 (2.0%)
Dermatological condition		159 (9.1%)
At least one unknown condition ^c^		4 (0.2%)
Mean number of medicines (SD, range)		2.4 (1.9, 1 to 16)
At least one medicine newly prescribed		500 (28.6%)
At least one	GI-system medicine	121 (6.9%)
	cardiovascular medicine	605 (34.7%)
	respiratory medicine	309 (17.7%)
	hypnotic or anxiolytic	50 (2.9%)
	antipsychotic	37 (2.1%)
	antidepressant	512 (29.3%)
	analgesic used for migraine	155 (8.9%)
	other analgesics	359 (20.6%)
	other CNS drug	88 (5,0%)
	infection medicine (antibiotics/HIV)	5 (0.3%)
	oral antidiabetics	65 (3.7%)
	insulin	186 (10.7%)
	other endocrine system drug	141 (8,1%)
	urinary tract disorder medicine	2 (0.1%)
	malignant disease or immunosuppression medicine	33 (1.9%)
	medicine for nutrition and blood, including herbal remedies	26 (1.5%)
	medicine used in rheumatic disease and gout	66 (3.8%)
	ophthalmic medicine	23 (1.3%)
	dermatologic medicine	84 (4.8%)
	corticosteroid with unspecified application	35 (2.0%)
	unknown medicine ^c^	377 (21.6%)
COVID-19-related factors		
Shielded		385 (22.1%)
Positive COVID-19 test		152 (8.7%)
Treatment for COVID-19		90 (5.2%)
Avoidance behaviour		
Avoiding/limiting contacts		1427 (81.7%)
Other protective measures (Face covering, washing hands)		1516 (86.8%)
No avoidance behaviour		89 (5.1%)

Data are presented as mean (SD, range) for continuous measures, and *n* (%) for categorical measures. BAME: Black, Asian and minority ethnic; CNS: central nervous system; CV: cardiovascular; GI: gastro-intestinal; LTC: long-term conditions patients reported to take medicines for; ^a^ in the third wave, more respondents did not report their post code (wave 1 *n*=13, wave 2 *n*=40, wave 3 *n*=140). The IMD score and rural or urban area were derived from postcode and therefore missingness varied between the waves; ^b^ in the third wave fewer people reported unknown when asked if they were exempt from prescription fee (wave 1 *n*=123, wave 2 *n*=108, wave 3 *n*=68); ^c^ unknown medicine/condition: specified by respondents as other or unknown, or response/drug name unrecognisable for analyst

The cohort had a mean (SD) age of 51.1 (17.2) and was 54.4% female, with a mean (SD) of 2.5 (1.9) LTCs, for which they were taking a mean (SD) of 2.4 (1.9) prescribed medicines. The most commonly prescribed categories of medicines were analgesics (*n*=514), antidepressants (*n*=512) and renin-angiotensin-aldosterone system agents and alpha-blockers (*n*=331). Of the 1746 respondents 385 (22.1%) were asked to shield and 152 (8.7%) had contracted COVID-19. More details on respondent characteristics are in Appendix 3.

### Access to medicines: ordering prescriptions from the general practice (see Appendix for full survey responses)

Only 124 (7.1%) respondents reported ordering their prescriptions from the GP in person. Most frequently reported alternative ordering methods were as follows: online via GP or other website, 590 (33.8%); ordered directly from a designated pharmacy via repeat dispensing, 357 (20.4%); telephoned GP, 248 (14.2%); GP app, 149 (8.5%); NHS app, 98 (5.6%); emailed GP, 77 (4.4%). Thirty-seven respondents (2.1%) reported that they were not collecting or receiving their medicines.

### Access to medicines: filling prescriptions from the pharmacy (see Appendix for full survey responses)

Most respondents (*n*=1048, 60.0%) reported collecting their prescriptions from the pharmacy themselves. The most frequently reported alternative collection methods were: picked up by friends and family: 245 (14.0%), delivered by the pharmacy: 223 (12.8%), online delivery: 130 (7.4%), picked up by formal carer or local support group: 74 (4.2%).

### Use of digital tools to support medicines management

Use of digital tools as a reminder to take medicines, to support ordering medicines, or both, was reported by 223 (12.8%), 81 (4.6%) and 50 (2.9%) respondents, respectively. This was a lower number of respondents than those that reported use of the GP or NHS app to order prescriptions, suggesting that these apps are not always perceived as tools to support medicines management. In total, 525 (30.1%) respondents reported using digital tools in both questions combined.

### Problems with access

No access to at least one medicine was reported by 182 (10.4%) respondents. The most frequently reported reasons for a problem with access are reported in Table 2.

**Table 2:** Problems with access to medicines

Variables	Total *N*=181^a^
I had to self-isolate	13 (7.2%)
I did not want to leave the house	26 (14.4%)
I did not want to use public transport	17 (9.4%)
The weather was bad	18 (9.9%)
I was not able to order a prescription by telephone	20 (11.0%)
I was not able to order a prescription by email/app	21 (11.6%)
I wasn’t allowed to go into the GP practice	25 (13.8%)
I don’t think my medicine is very important and did not want to bother my GP	12 (6.6%)
The GP practice did not allow ordering by phone	24 (13.3%)
Only online orders available at my GP and I don’t have internet access	17 (9.4%)
Only online orders available at my GP and I do not know how to order online	18 (9.9%)
Only online orders available at my GP and I do not want to use them	22 (12.2%)
I did not get an appointment at my GP practice	12 (6.6%)
Pharmacy was closed	15 (8.3%)
Queue was too long at the pharmacy	17 (9.4%)
Pharmacy did not do home deliveries	17 (9.4%)
People weren’t social distancing in the queue, and I did not feel safe	17 (9.4%)
The pharmacy was too far away	19 (10.5%)
Medicines were out of stock or unavailable	32 (17.7%)
Other	25 (13.8%)

Data are presented as n (%). ^a^ Number of respondents reporting no access to at least one medicine

### Actions taken to resolve access problems

Of the people specifically reporting access problems and asked to state how they solved these, GP access was achieved via friends/relatives/local action group (24), email (12), app (15), telephone (27), or website (26), or by breaking their isolation (9). Problems with pharmacy access were solved via friends/relatives/local action group (41), pharmacy delivery (32), online delivery (32), using a different pharmacy (37) or by breaking their isolation (20). See Appendix 2 for full survey responses.

Over half of the cohort reported taking at least one non-prescription medicine as substitute: 1048 (60.0%), and nearly a quarter of patients requested emergency supply from pharmacy for at least one medicine: 409 (23.4%). These numbers seem high and warrant further investigation through interview to assess response validity and if there is any association with the COVID-19 pandemic.

Respondents reported using A&E [Accident and Emergency] (16), an out-of-hours GP (17), a walk-in centre (17) or NHS helpline (18). Twenty-three respondents reported missing their prescription due to access issues.

### Adherence

Of 1764 respondents, 394 (22.6%) reported non-adherence to at least one medicine in the previous 7 days, with a mean (SD) of 6.1 (8.3) doses missed by people reporting non-adherence. Adherence ranged from 69% in central nervous system medicines to 84% in cardiovascular medicines (Supplementary Table 5). Mean (SD) scores for potential reasons reported for non-adherence were: physical capability: 4.9 (3.5), psychological capability: 5.4 (3.5), physical opportunity: 5.3 (3.5), social opportunity: 5.1 (3.5), reflective motivation: 5.7 (3.4) and automatic motivation: 7.0 (3.3). Results of the Friedman test showed that differences in the mean scores between the different reasons for non-adherence were statistically significant (Q=130.81, *p* < 0.000). A one-way repeated measures analysis of variance (ANOVA) was not possible, because the assumptions of normality and sphericity were violated.

### Factors affecting adherence

Table 3 reports the univariate and multivariate analysis of factors associated with adherence. All factors that reached statistical significance (*p*<0.05) in the univariate analysis were included in the multivariate analysis, and those that remained significant are indicated in bold.

**Table 3:** Factors associated with non-adherence (*N*=1746)

Independent variables	*N* (%) all	*N* (%) non-adherent	OR (95% CI)	Adjusted OR (95% CI) ^a^
Age				
16–24	157 (9.0%)	82 (52.2%)	1	1
25–34	221 (12.7%)	100 (45.2%)	0.71 [0.46–1.11]	0.64 [0.35, 1.19]
35–44	234 (13.4%)	82 (35.0%)	0.42 [0.27–0.65]	**0.39 [0.21, 0.73]**
45–54	331 (19.0%)	77 (23.3%)	0.26 [0.17–0.40]	**0.39 [0.22, 0.71]**
55–64	319 (18.3%)	29 (9.1%)	0.07 [0.04–0.13]	**0.15 [0.07, 0.30]**
65+	484 (27.7%)	24 (5.0%)	0.04 [0.02–0.07]	**0.14 [0.07, 0.31]**
Gender				
Male	794 (45.5%)	226 (28.5%)	1	1
Female	949 (54.4%)	167 (17.6%)	0.53 [0.41–0.68]	**0.60 [0.43, 0.85]**
Unknown	3 (0.2%)	1 (33.3%)	1.33 [0.11–15.60]	1.93 [0.88, 4.25]
Ethnicity				
White	1529 (87.6%)	303 (19.8%)	1	1
Black, Asian and minority ethnic populations	148 (8.5%)	65 (43.9%)	3.63 [2.47–5.34]	1.14 [0.71, 1.82]
Unknown	69 (4.0%)	26 (37.7%)	3.70 [2.09–6.53]	1.61 [0.79, 3.30]
Region				
Scotland	170 (9.7%)	38 (22.4%)	1.59 [0.86–2.95]	1.93 [0.88, 4.25]
North East	56 (3.2%)	10 (17.9%)	1.08 [0.43–2.68]	1.10 [0.46, 2.64]
Yorkshire/Humber	140 (8.0%)	29 (20.7%)	1.33 [0.69–2.56]	1.58 [0.74, 3.40]
North West	234 (13.4%)	70 (29.9%)	2.13 [1.20–3.77]	1.75 [0.91, 3.36]
East Midlands	130 (7.4%)	27 (20.8%)	1.32 [0.68–2.56]	1.16 [0.53, 2.54]
West Midlands	123 (7.0%)	22 (17.9%)	1.13 [0.57–2.25]	0.86 [0.39, 1.94]
South East	228 (13.1%)	46 (20.2%)	1.19 [0.65–2.17]	1.39 [0.66, 2.91]
East of England	203 (11.6%)	43 (21.2%)	1.37 [0.75–2.52]	1.52 [0.73, 3.13]
Greater London	171 (9.8%)	52 (30.4%)	2.28 [1.26–4.15]	1.48 [0.71, 3.08]
Wales	162 (9.3%)	24 (14.8%)	1	1
West	98 (5.6%)	21 (21.4%)	1.25 [0.62–2.53]	1.35 [0.58, 3.14]
Northern Ireland	31 (1.8%)	12 (38.7%)	4.28 [1.66–11.08]	2.28 [0.79, 6.60]
Socioeconomic group ^b^				
1	203 (11.6%)	44 (21.7%)	1	1
2	248 (14.2%)	57 (23.0%)	1.09 [0.67–1.75]	1.37 [0.75, 2.49]
3	453 (25.9%)	96 (21.2%)	0.96 [0.62–1.48]	1.26 [0.73, 2.16]
4	395 (22.6%)	102 (25.8%)	1.20 [0.78–1.85]	1.43 [0.83, 2.47]
5	110 (6.3%)	48 (43.6%)	2.88 [1.69–4.93]	**2.49 [1.22, 5.09]**
Student	26 (1.5%)	9 (34.6%)	1.83 [0.73–4.56]	0.51 [0.15, 1.71]
Retired	120 (6.9%)	4 (3.3%)	0.15 [0.05–0.45]	0.78 [0.19, 3.25]
Unemployed	191 (10.9%)	34 (17.8%)	0.73 [0.43–1.24]	1.07 [0.50, 2.32]
Index of multiple deprivation				
Q1 most deprived	530 (30.4%)	123 (23.2%)	1	1
Q2	294 (16.8%)	71 (24.1%)	0.91 [0.63–1.33]	1.02 [0.63, 1.65]
Q3	251 (14.4%)	56 (22.3%)	0.93 [0.62–1.40]	1.06 [0.65, 1.75]
Q4	250 (14.3%)	55 (22.0%)	0.81 [0.55–1.20]	1.40 [0.86, 2.31]
Q5 least deprived	230 (13.2%)	39 (17.0%)	0.51 [0.33–0.78]	0.81 [0.47, 1.41]
Unknown	191 (10.9%)	50 (26.2%)	1.06 [0.70–1.61]	0.87 [0.51, 1.50]
Working	863 (49.4%)	291 (33.7%)	4.65 [3.54–6.11]	**1.81 [1.17, 2.81]**
Household size				
1 person	388 (22.2%)	51 (13.1%)	1	1
2 persons	687 (39.3%)	94 (13.7%)	1.22 [0.81–1.83]	1.18 [0.73, 1.92]
3+ with children	428 (24.5%)	195 (45.6%)	6.40 [4.33–9.48]	1.25 [0.77, 2.05]
3+ without children	243 (13.9%)	54 (22.2%)	2.13 [1.33–3.40]	1.08 [0.62, 1.87]
Factors associated with COVID-19				
Shielded	385 (22.1%)	130 (33.8%)	2.10 [1.59–2.78]	0.78 [0.51, 1.18]
Positive COVID-19 test	152 (8.7%)	74 (48.7%)	3.91 [2.67–5.73]	0.91 [0.56, 1.45]
Factors related to access to medicine				
Times with no access to a medicine	0.2 (0.8)	0.6 (1.3)	2.80 [1.53–5.11]	**1.50 [1.18, 1.89]**
Times non-prescription medicines were used as substitutes	3.3 (2.6)	3.8 (2.4)	1.11 [1.06–1.16]	1.04 [0.98, 1.12]
Times immediate supply from pharmacy was required	1.6 (1.4)	2.6 (2.0)	2.03 [1.78–2.30]	**1.38 [1.22, 1.57]**
Exempt from prescription fee	299 (17.1%)	71 (23.7%)	0.52 [0.40–0.67]	1.11 [0.72, 1.72]
Suitable public transport available?	1295 (74.2%)	307 (23.7%)	1.35 [1.00–1.82]	0.78 [0.52, 1.17]
Help taking medicines at home?	273 (15.6%)	7 (18.4%)	4.63 [3.42–6.27]	1.32 [0.85, 2.06]
Use of digital tools to support medicines management				
Yes, to order or as reminder to take medicines ^c^	525 (30.1%)	203 (38.7%)	1.76 [1.56–1.98]	1.37 [0.99, 1.88]
Drug/LTC-related factors				
Number of medicines	2.4 (1.9)	2.0 (1.8)	0.82 [0.72–0.92]	0.91 [0.79, 1.04]
Number of LTCs newly diagnosed since March 2020	0.4 (0.9)	0.8 (1.2)	2.17 [1.78–2.65]	1.16 [0.94, 1.43]
Number of drugs newly prescribed since March 2020	0.6 (1.2)	0.9 (1.3)	1.31 [1.19–1.45]	1.07 [0.90, 1.28]
At least one cardiovascular medicine	605 (34.7%)	61 (10.1%)	0.24 [0.17–0.33]	**0.60 [0.38, 0.95]**
At least one respiratory medicine	309 (17.7%)	54 (17.5%)	0.70 [0.49–0.99]	0.63 [0.39, 1.00]
Analgesics for migraine	514 (29.4%)	151 (29.4%)	1.71 [1.32–2.23]	1.25 [0.77, 2.03]
Insulin	186 (10.7%)	16 (8.6%)	0.27 [0.15–0.49]	**0.49 [0.24, 1.01]**
Thyroid and antithyroid medicines	116 (6.6%)	9 (7.8%)	0.28 [0.13–0.60]	**0.32 [0.11, 0.93]**
At least one medicine used in rheumatic disease and gout	23 (1.3%)	2 (3.0%)	0.13 [0.03–0.55]	**0.10 [0.02, 0.48]**
At least on anxiolytic or hypnotic medicine	50 (2.9%)	24 (48.0%)	3.62 [1.98–6.63]	2.13 [0.96, 4.70]

^a^Odds ratio adjusted for age, gender ethnicity, region, socioeconomic group, IMD, work status, household size, new LTCs, number of medicines, exemption from prescription fee, suitable public transport, help taking medicines at home, use of digital tools by type, shielding status, positive COVID-19 test, CV-medicine, respiratory medicine, analgesic, insulin, thyroid medicine, medicine for rheumatic disease or gout [significance indicated in bold]; ^b^ 1: semi or unskilled manual worker, 2: skilled manual worker, 3: supervisory or junior managerial or professional or administrator, 4: intermediate managerial or professional or administrative, 5: higher managerial or professional or administrative; ^c^ Use of digital tools reported for question 15 “How did you order your prescription at your GP the last time you renewed your prescription?” and question 26 “Did you use any apps or digital tools to support you to take your medicine?”

Younger, male, or working respondents, and those in higher managerial/professional/administrative jobs (Socioeconomic group 5) were less adherent. Taking medicines for cardiovascular, thyroid or rheumatic disease or gout or insulin use was associated with better adherence.

If a respondent had no access to medicines or required immediate supply from the pharmacy, the odds of being non-adherent increased each time this occurred by 50% (adj. OR 1.50 [95% CI 1.18, 1.89]) and 38% (adj. OR 1.38 [95% CI 1.22, 2.57]), respectively.

In a supplementary analysis, we analysed the use of digital tools as a categorical instead of a binary variable in the regression model (see table 7, Supplementary Appendix). The model fit however was better for the model including digital tools as a binary variable, which was used as the main analysis. In the supplementary analysis, the use of digital tools to help as a reminder to take medicines was associated with poorer adherence (adj. OR 1.73 [95% CI: 1.11,2.71]).

## Discussion

### Main findings

Results from this UK-wide survey of adults taking medicines for LTCs suggest that just under a quarter of people report non-adherence to one or more prescribed medicines. Our findings suggest no significant association of positive COVID-19 tests or shielding at home with adherence. Access problems, on the other hand, were common during the COVID-19 pandemic and were identified as a key driver of non-adherence, along with being younger, male, being in the highest socioeconomic group and working. Access problems reported were mostly directly or indirectly related to the COVID-19 pandemic. It is difficult to compare adherence levels with pre-COVID-19 levels, due to cohort and adherence measurement differences between studies. However, comparison with adherence studies in UK cohorts pre-COVID-19 in three of the largest therapeutic areas present in our cohort (hypertension, diabetes and depression) suggests that adherence across our cohort did not appear to be demonstrably different. Pre-COVID adherence in people taking antihypertensives in the UK was reported at about 80% (Kurdi et al. 2017), compared with 86.7% in this study. Pre-COVID adherence in people taking oral diabetes medication in the UK was reported to range from 60% (Tunceli et al. 2015) to 87% (Farmer et al. 2015), with 71% adherence to insulin) (Donnelly et al. 2007) compared with 74.6% for the two categories combined in this study. Pre-COVID adherence in people taking anti-depressants in the UK was reported at about 69% (Hafferty et al. 2019), compared with 72.7% in this study.

### What is already known on this topic

About 25% of medicines prescribed for LTCs are probably not taken as directed, although reported medicines adherence ranges from 4 to 100% due to a wide variety of patient populations, diseases and medicines considered, compounded by varying study designs, definitions and measurement of adherence (DiMatteo 2004). Medicines adherence can be influenced by patient- and disease-related factors such as socio-demographic characteristics, type and severity of illness, number of medicines and regimen complexity, introduction of new medicines, fear/experience of side-effects, and system-related factors such as out-of-pocket costs, access to, and relationship with healthcare providers (Elliott 2006, 2008). Medicines-taking is a highly contextualised behaviour where barriers such as disruption of routines, forgetting, insufficient social support and barriers to accessing prescriptions or medicines can reduce adherence (Horne et al. 2005).

We carried out a literature search and found four studies looking at medicines-taking and adherence during the COVID-19 pandemic: one from the UK (Garfield et al. (2021), India (Subathra et al. 2021), China (Wang et al. 2021) and Portugal (Midao et al. 2022). Garfield et al. (2021) interviewed 50 UK patients shielding during the COVID-19 pandemic about their medicines management. Midao et al. (2022) conducted a cross-sectional online survey in a Portuguese patient cohort. Both studies identified access issues as key problems that emerged with the COVID-19 pandemic. Garfield et al. (2021) highlighted patients’ anxiety around accessing medications, and Midao et al. (2022) report the fear of leaving home as a reason for non-adherence. Most of the respondents in the Portuguese cohort did not think that COVID-19 impacted their adherence.

### What this study adds

This large survey was the first study to examine the effects of COVID-19 on medicines-taking in a sample representative of the UK population with regard to gender, age, socioeconomic group and household size. Reported LTCs and medicines were broadly representative of those in the general UK population. An observational study using GP data between 2012 and 2016 identified hypertension, depression/anxiety and chronic pain as the most commonly prescribed LTCs (Cassell et al. 2018). These were the most commonly reported LTCs in our cohort. With regards to medicines, the most frequently dispensed medicines by BNF chapter according to the Prescription Cost Analysis (PCA) 2020 were central nervous system (CNS), cardiovascular and endocrine system medicines, which reflects the proportions of medicines reported in this survey (NHS Digital 2021). Only gastrointestinal (GI) system drugs were comparably less commonly reported in this dataset than in the PCA, which may warrant further investigation.

In common with other studies, we found that age, working status and gender were associated with adherence. Increasing age was independently associated with higher adherence, which reflects other work that once cognitive decline and numbers of medicines are controlled for, older adults are generally more adherent than younger adults until they become very elderly (Viller et al. 1999; Gast and Mathes 2019; Kim et al. 2019), although the evidence varies between diseases (Hughes 2004; Gast and Mathes 2019). This may be due to greater severity of illness increasing awareness about their health status and the fact that younger people may have work commitments and childcare responsibilities that contribute to reduced adherence. This latter factor is also suggested to be important given that respondents reporting they were “working” were less adherent. Treatment burden in working people has been reported to lead to non-adherence (Trakoli 2021). In general, the evidence suggests an increase in adherence with socioeconomic status, which wasn’t evident in this study, measured either by socioeconomic group or deprivation status. This may be partly attributable to wide access to free prescription medicines in the UK confounding the effect of these factors. Interestingly, in our study, being in the highest socioeconomic group was independently associated with poorer adherence. Whilst this might be an anomaly in the data, this observation warrants further investigation to assess whether the COVID-19 pandemic affected this group differently, for example, this group might have been more likely to be working from home (Office for National Statistics 2022).

Being male was significantly associated with reduced adherence in our cohort. More generally, the impact of gender is uncertain, a recent review of reviews reporting conflicting effect directions within and between systematic reviews, so it is difficult to postulate why this effect was seen, other than the restrictions of the COVID-19 pandemic exacerbating men’s reduced tendency to healthcare-seeking behaviour (Gast and Mathes 2019; Manteuffel et al. 2014; Armitage et al. 2021).

Our findings identified access problems as a key driver of non-adherence. As we have described above, the results from our study did not suggest poorer adherence compared with pre-COVID levels. However, reported reasons for non-adherence, when it occurred, seemed to be dominated by access problems, more than in pre-COVID studies. For example, in diabetes, younger age and severity of illness dominate reasons for poor adherence (Tunceli et al. 2015) in a pre-COVID UK cohort. In hypertension, younger age, presence of physical comorbidities and socioeconomic deprivation dominate reasons for poor adherence (Kurdi et al. 2017) in a pre-COVID UK cohort. In depression, being female, presence of physical comorbidities and severity of illness dominate reasons for poor adherence (Hafferty et al. 2019) in a pre-COVID UK cohort. In our study, severity of illness, usually seen to be associated with adherence, did not reach significance.

Many of the reported reasons for access problems were likely consequences of COVID-19-related avoidance behaviour of individuals (e.g. I had to self-isolate, I did not want to leave the house). In addition to these general COVID-19 factors, restrictions limiting face-to-face access to GP services and transitions to online service provision during the pandemic (e.g. I was not allowed to go into the GP practice, only online orders available, I did not get an appointment at my GP) seemed to affect access to medicines in our cohort. Furthermore, pharmacy access and medicines supply seemed adversely affected due to COVID-related factors (e.g. queue was too long, medicines out of stock, pharmacy didn’t do home deliveries). Over half of our cohort reported substituting a non-prescription medicine or requesting an emergency supply at the pharmacy, and further work is needed to establish whether this was due to problems with obtaining a GP appointment or prescription, or with supply issues at the pharmacy, what therapeutic areas were most affected, and whether this was an occasional dose or more frequent substitution.

Given that adherence levels did not seem to be lower than pre-COVID, it may be that respondents were resourceful in getting around access issues, some using remote or digital services for the first time to order prescriptions or obtain medicines, others using friends and family to collect medicines or using a different pharmacy. Over half of respondents reported resorting to substituting non-prescription medicines, requesting emergency supplies at the pharmacy, a small number using A&E or walk-in centres to obtain medicines, and a few reporting that they didn’t get their medicine at all. These “last-ditch” approaches are likely to have resulted in adverse health consequences for the patient, and resource use consequences for the health service. Habit-forming is an important aspect of medicines adherence (Fontanet et al. 2021), and this is likely to have been hampered by COVID-19 restrictions.

Our study provides insights into why people were non-adherent. Some patients experienced practical barriers to adherence (such as lockdown restrictions, extra practical burden associated with accessing GPs and community pharmacies, medicines availability and disrupted routines) or perceptual barriers (such as mental burden, concerns about contracting COVID, or not wanting to “burden” the GP/NHS). We know that perception of treatment burden leads to non-adherence (Ibrahim et al. 2021), so the extra burden associated with accessing medicines during COVID-19 restrictions is likely to have contributed to reduced adherence. Our findings suggest that people were generally lacking in capabilities and opportunities, but that disruptions to habits (automatic motivation) were the major reason for non-adherence. This supports the qualitative findings from Garfield et al. (2021), where habit disruption was reported. This is consistent with our related work on adherence to government COVID-19-related instructions showing that helping people develop new habits is a good step forward in supporting adherence (Armitage et al. 2021).

A key change during the COVID-19 pandemic was a rapid move from primarily face-to-face interactions to remote access (Murphy et al. 2021; Neves et al. 2021a). This change was more evident for GP access, with only 7.1% of respondents reporting collecting prescriptions in person and 60% of people reporting still collecting their medicine from the pharmacy in person. We know that people with LTCs tend to prefer face-to-face interaction (Iglesias Urrutia et al. 2022), and so this transition is likely to have been problematic for some people. During the lockdown period, around 61% of GP appointments were conducted via telephone, with 4% via video (Royal College of General Practitioners 2020a, b). Our survey revealed multiple methods being offered by GPs to order prescriptions.

About a third (30.1%) of respondents reported the use of digital tools to support chronic medicines management, such as reminders to take medicines or to support ordering of new medicines. The use of these was, however, not independently associated with better adherence in our main analysis and was actually linked to poorer adherence in a supplementary analysis. It is probable that people struggling with their medicines, and with poor adherence, might be more likely to have digital tools recommended to them. While apps have been proven to improve medicines adherence in LTCs in randomised controlled trials (RCTs) (Armitage et al. 2020), there are many apps available supported by little evidence, and it is unclear how effective or appropriate the apps were that were used by the respondents in our cohort. Furthermore, while respondents indicated they had an app, it is not known how often they used it. The findings suggest that there is a need to support people with LTCs to find and use high-quality, evidence-based digital tools that can support their medicines-taking, if they wish to do so. There is an increasing expectation that delivering services remotely (such as telephone/video consultations) (Lynnerup et al. 2022), and digital health interventions (such as self-management apps or ordering prescriptions through a website or app) can help people in managing their health (Cresswell et al. 2021). This assumes that people with LTCs have access to, and the ability to use, remote and digital technologies that work well across the board to support medicines use (Royal College of General Practitioners 2020a, b; Were et al. 2022).

In summary, the overall adherence of the cohort did not appear to be much lower than what might be expected in people with LTCs. COVID-19-related behaviour and restrictions appeared to add to, dominate or replace known barriers to medicines-taking. People experienced reduced face-to-face contact with healthcare professionals leading to increased reliance on social networks and remote/digital routes to order and fill prescriptions. With reduced face-to-face contact with general practice, there was a bewildering array of remote methods for ordering and filling prescriptions, some of these systems introduced “overnight”. People seemed to be fairly resilient in finding ways to access medicines, but some had problems with some remote/digital methods. The transformation of primary care during the COVID-19 pandemic (Neves et al. 2021b) means that some services may never revert to pre-COVID-19 models. Our cohort reported that problems with using remote and digital services served as a barrier to medicines access, so this mismatch in provider and patient expectations needs to be addressed to avoid access problems. A recent review of policy around virtual and remote healthcare suggests that work is needed to address “shortcomings exposed during COVID-19” (Neves et al. 2021c). We suggest that more needs to be done to enhance people’s capabilities and opportunities, if necessary, beyond the digital sphere through home visits, health visitors or mail, or targeting face-to-face consultations where a benefit is likely to be greatest.

### Limitations of this study

Use of a self-report measure for adherence is a potential weakness. We had no access to other methods, such as prescription-filling data. Self-report tends to return higher rates of medication adherence than some objective measures (Foley et al. 2021), due to social desirability and memory bias. However, when patients report that they have been non-adherent, these accounts are generally accurate (Choo et al. 1999), and patient-reported adherence correlates with objective clinical measures (Murri et al. 2000; DiMatteo et al. 2002; Makela et al. 2013). We minimised biases through anonymous survey interview (Butler et al. 2004), made efforts to normalise non-adherence by recognising the challenges of taking regular medications and asked about missed doses only in the week prior to data collection, to optimise recall (Lehmann et al. 2014).

Information on reasons for access problems and actions taken to resolve them, as well as COVID-infection treatment details were not included in the logistic regression analysis because patient numbers for each response option were small and these questions were only answered by a subset of the population, that is, those with access problems and a positive COVID-19 infection, respectively. Hence, missingness for these variables was by design almost 90% and 80%, respectively.

Nearly three-quarters of patients did not know whether they were eligible for a prepayment certificate (PPC, 12 months £108.10, 2022) so we were not able to use this parameter in our regression analysis. (National Health Service). This is a useful finding as it supports the notion that there is generally poor awareness of PPCs (Mason 2018), suggesting that more should be done to make people managing LTCs with multiple medicines aware, especially considering that prescription co-payments are strongly correlated with poorer adherence (Sinnott et al. 2013; Gast and Mathes 2019).

Fifty-two respondents in our cohort reported COVID-19 hospitalisations over the pandemic period, and 46 (88.5%) of this group reported non-adherence to medicines in the previous 7 days. It is not clear why and requires further qualitative work to determine whether this is a true association, or whether respondents misunderstood the question.

The timing of our survey was over a year into the pandemic, after two strict lockdowns and subsequent easing, which will have affected COVID-19 avoidance behaviour and restrictions. Therefore, it is not clear whether the barriers and behaviours being reported were during strict lockdowns or when restrictions had eased. However, we know that adherence behaviour was that reported for the previous 7 days, in the summer of 2021, so people had had time to adjust to initial changes.

We have not reported subgroup analysis due to sample size restrictions. It is possible that some of these phenomena will have a different appearance or prevalence in subgroups such as specific illnesses, older people, or people with multi-morbidities.

## Conclusions

This study reported suboptimal medicines adherence in a representative cohort of adults with LTCs. COVID-19 related behaviour and restrictions appeared to add to, dominate or replace some known barriers to medicines-taking. Our study provides insight into the level of reduced face-to-face contact with general practice, during COVID-19, in contrast to more face-to-face contact with community pharmacies. Our study provides a detailed insight into how people with LTCs are accessing prescribers to obtain medicines, and how they subsequently access the medicines themselves. There was a bewildering array of remote methods for ordering and filling prescriptions, some of these systems introduced “overnight”. People seemed to be fairly resilient in finding ways to access medicines, but some had problems with some remote/digital methods, which may have contributed to reduced adherence. Given that remote and digital interventions and services are here to stay, successful implementation depends upon tailoring interventions to the unique characteristics of patients, disease conditions, and treatment regimens and supporting people to engage effectively with the digital services provided.

## Supplementary information

ESM 1 (DOCX 75 kb)

## Data Availability

The datasets generated and/or analysed during the current study are not publicly available due to data sharing agreements but are available from the corresponding author on reasonable request.

## List of abbreviations

BNF: British National Formulary
CNS: Central nervous system
COM-B: Capability, opportunity and motivation model of behaviour
GP: General practitioner
IMD: Index of multiple deprivation
LTC: Long-term conditions
NHS: National Health Service
OR: Odds ratio
SD: Standard deviation
UK: United Kingdom

## References

[CR1] Armitage LC, Kassavou A, Sutton S (2020). Do mobile device apps designed to support medication adherence demonstrate efficacy? A systematic review of randomised controlled trials, with meta-analysis. BMJ Open.

[CR2] Armitage CJ, Keyworth C, Leather JZ, Byrne-Davis L, Epton T (2021). Identifying targets for interventions to support public adherence to government instructions to reduce transmission of SARS-CoV-2. BMC Public Health.

[CR3] Butler JA, Peveler RC, Roderick P, Horne R, Mason JC (2004). Measuring compliance with drug regimens after renal transplantation: comparison of self-report and clinician rating with electronic monitoring. Transplantation.

[CR4] Cabinet Office (2021) COVID-19 Response: Summer 2021. G. UK. https://www.gov.uk/government/publications/covid-19-response-summer-2021-roadmap/covid-19-response-summer-2021

[CR5] Cassell A, Edwards D, Harshfield A, Rhodes K, Brimicombe J, Payne R, Griffin S (2018). The epidemiology of multimorbidity in primary care: a retrospective cohort study. Br J Gen Pract.

[CR6] Choo PW, Rand CS, Inui TS, Lee ML, Cain E, Cordeiro-Breault M, Canning C, Platt R (1999). Validation of patient reports, automated pharmacy records, and pill counts with electronic monitoring of adherence to antihypertensive therapy. Med Care.

[CR7] Chowdhury R, Khan H, Heydon E, Shroufi A, Fahimi S, Moore C, Stricker B, Mendis S, Hofman A, Mant J, Franco OH (2013). Adherence to cardiovascular therapy: a meta-analysis of prevalence and clinical consequences. Eur Heart J.

[CR8] Clifford S, Barber N, Elliott R, Hartley E, Horne R (2006). Patient-centred advice is effective in improving adherence to medicines. Pharm World Sci.

[CR9] Cresswell K, Williams R, Sheikh A (2021). Using cloud technology in health care during the COVID-19 pandemic. Lancet Digital Health.

[CR10] Cutler RL, Fernandez-Llimos F, Frommer M, Benrimoj C, Garcia-Cardenas V (2018). Economic impact of medication non-adherence by disease groups: a systematic review. BMJ Open.

[CR11] Digital NHS (2021) Prescription Cost analysis (PCA)- England 2020/2021 - Calendar year 2020 - National level. NHSBSA https://www.nhsbsa.nhs.uk/statistical-collections/prescription-cost-analysis-england/prescription-cost-analysis-england-202021

[CR12] DiMatteo MR (2004). Variations in patients' adherence to medical recommendations: a quantitative review of 50 years of research. [see comment]. Med Care.

[CR13] DiMatteo MR, Giordani PJ, Lepper HS, Croghan TW (2002). Patient adherence and medical treatment outcomes: a meta-analysis. Med Care.

[CR14] Donnelly LA, Morris AD, Evans JM (2007). Adherence to insulin and its association with glycaemic control in patients with type 2 diabetes. Q J Med.

[CR15] Elliott RA (2006). Poor Adherence to Anti-inflammatory Medication in Asthma. Disease Manag Health Outcomes.

[CR16] Elliott RA (2008). Poor Adherence to Medication in Adults with Rheumatoid Arthritis. Disease Manag Health Outcomes.

[CR17] Elliott RA, Boyd MJ, Tanajewski L, Barber N, Gkountouras G, Avery AJ, Mehta R, Davies JE, Salema NE, Craig C, Latif A, Waring J, Chuter A (2020). 'New Medicine Service': supporting adherence in people starting a new medication for a long-term condition: 26-week follow-up of a pragmatic randomised controlled trial. BMJ Qual Saf.

[CR18] Farmer AJ, Rodgers LR, Lonergan M, Shields B, Weedon MN, Donnelly L, Holman RR, Pearson ER, Hattersley AT, Consortium F T M (2015). Adherence to Oral Glucose-Lowering Therapies and Associations With 1-Year HbA1c: A Retrospective Cohort Analysis in a Large Primary Care Database. Diabetes Care.

[CR19] Foley L, Larkin J, Lombard-Vance R, Murphy AW, Hynes L, Galvin E, Molloy GJ (2021). Prevalence and predictors of medication non-adherence among people living with multimorbidity: a systematic review and meta-analysis. BMJ Open.

[CR20] Fontanet CP, Choudhry NK, Wood W, Robertson T, Haff N, OranR SES, Kim E, Hanken K, Barlev RA, Lauffenburger JC, Feldman CH (2021). Randomised controlled trial targeting habit formation to improve medication adherence to daily oral medications in patients with gout. BMJ Open.

[CR21] Garfield S, Wheeler C, Boucher C, Etkind M, Lloyd J, Norton J, Ogunleye D, Taylor A, Williams M, Grimes T, Kelly D, Franklin BD (2021). Medicines management at home during the COVID-19 pandemic: a qualitative study exploring the UK patient/carer perspective. Int J Pharm Pract.

[CR22] Gast A, Mathes T (2019). Medication adherence influencing factors-an (updated) overview of systematic reviews. Syst Rev.

[CR23] Hafferty JD, Wigmore EM, Howard DM, Adams MJ, Clarke T-K, Campbell AI, Macintyre DJ, Nicodemus KK, Lawrie SM, Porteous DJ, Mcintosh AM (2019). Pharmaco-epidemiology of antidepressant exposure in a UK cohort record-linkage study. J Psychopharmacol.

[CR24] Ho PM, Rumsfeld JS, Masoudi FA, McClure DL, Plomondon ME, Steiner JF, Magid DJ (2006). Effect of medication nonadherence on hospitalization and mortality among patients with diabetes mellitus. Arch Intern Med.

[CR25] Hong JS, Sheriff R, Smith K, Tomlinson A, Saad F, Smith T, Engelthaler T, Phiri P, Henshall C, Ede R, Denis M, Mitter P, D'Agostino A, Cerveri G, Tomassi S, Rathod S, Broughton N, Marlowe K, Geddes J, Cipriani A (2021). Impact of COVID-19 on telepsychiatry at the service and individual patient level across two UK NHS mental health Trusts. Evid Based Ment Health.

[CR26] Horne R, Weinman J, Barber N, Elliott R, Morgan M (2005). Concordance, adherence and compliance in medicine taking: a scoping exercise.

[CR27] Hughes CM (2004). Medication non-adherence in the elderly: how big is the problem?. Drugs Aging.

[CR28] Ibrahim KM, Schommer JC, Morisky DE, Rodriguez R, Gaither C, Snyder M (2021) The Association between Medication Experiences and Beliefs and Low Medication Adherence in Patients with Chronic Disease from Two Different Societies: The USA and the Sultanate of Oman. Pharmacy (Basel) 9(1). 10.3390/pharmacy9010031 10.3390/pharmacy9010031 PMC7931077 33546425

[CR29] Iglesias Urrutia CP, Erdem S, Birks YF, Taylor SJC, Richardson G, Bower P, van den Berg B, Manca A (2022). People's preferences for self-management support. Health Serv Res.

[CR30] Keyworth C, Epton T, Goldthorpe J, Calam R, Armitage CJ (2020). Acceptability, reliability, and validity of a brief measure of capabilities, opportunities, and motivations (COM-B). Br J Health Psychol.

[CR31] Keyworth C, Epton T, Byrne-Davis L, Leather JZ, Armitage CJ (2021). What challenges do UK adults face when adhering to COVID-19-related instructions? Cross-sectional survey in a representative sample. Prev Med.

[CR32] Kim SJ, Kwon OD, Han EB, Lee CM, Oh SW, Joh HK, Oh B, Kwon H, Cho B, Choi HC (2019). Impact of number of medications and age on adherence to antihypertensive medications: A nationwide population-based study. Medicine (Baltimore).

[CR33] Kurdi AI, Chen L-C, Elliott RA (2017). Exploring factors associated with patients’ adherence to antihypertensive drugs among people with primary hypertension in the United Kingdom. J Hypertens.

[CR34] Lehmann A, Aslani P, Ahmed R, Celio J, Gauchet A, Bedouch P, Bugnon O, Allenet B, Schneider MP (2014). Assessing medication adherence: options to consider. Int J Clin Pharm.

[CR35] Lynnerup C, Norreslet M, Graabaek T (2022). Attitudes towards video communication for New Medicine Service at community pharmacies - A qualitative pilot study. Explor Res Clin Soc Pharm.

[CR36] Makela MJ, Backer V, Hedegaard M, Larsson K (2013). Adherence to inhaled therapies, health outcomes and costs in patients with asthma and COPD. Respir Med.

[CR37] Manteuffel M, Williams S, Chen W, Verbrugge RR, Pittman DG, Steinkellner A (2014). Influence of patient sex and gender on medication use, adherence, and prescribing alignment with guidelines. J Women's Health (Larchmt).

[CR38] Mason C (2018) Revealed: 800,000 could have saved with a prescription 'season ticket' last year. MoneySavingExpert. online. https://www.moneysavingexpert.com/news/2018/01/revealed-800000-could-have-saved-with-a-prescription-season-ticket-last-year/

[CR39] Midao L, Almada M, Carrilho J, Sampaio R, Costa E (2022) Pharmacological Adherence Behavior Changes during COVID-19 Outbreak in a Portugal Patient Cohort. Int J Environ Res Public Health 19(3). 10.3390/ijerph19031135 10.3390/ijerph19031135 PMC8835016 35162159

[CR40] Mongkhon P, Ashcroft DM, Scholfield CN, Kongkaew C (2018). Hospital admissions associated with medication non-adherence: a systematic review of prospective observational studies. BMJ Qual Saf.

[CR41] Murphy M, Scott LJ, Salisbury C, Turner A, Scott A, Denholm R, Lewis R, Iyer G, Macleod J, Horwood J (2021). Implementation of remote consulting in UK primary care following the COVID-19 pandemic: a mixed-methods longitudinal study. Br J Gen Pract.

[CR42] Murri R, Ammassari A, Gallicano K, Luca AD, Cingolani A, Jacobson D, Wu AW, Antinori A (2000). Patient-Reported Nonadherence to HAART Is Related to Protease Inhibitor Levels. J Acquir Immune Defic Syndr.

[CR43] National Institute for Health and Care Excellence (2014) Behaviour change: individual approaches. Public health guideline. https://www.nice.org.uk/guidance/ph49/resources/behaviour-change-individual-approaches-pdf-1996366337989

[CR44] Neves AL, Li E, Serafini A, Jimenez G, Lingner H, Koskela TH, Hoffman RD, Collins C, Petek D, Claveria A, Tsopra R, Irving G, Gusso G, O'Neill B, Hoedebecke K, Espitia SM, Ungan M, Nessler K, Lazic V, Laranjo L, Memarian E, Fernandez MJ, Ghafur S, Fontana G, Majeed A, Car J, Darzi A (2021). Evaluating the Impact of COVID-19 on the Adoption of Virtual Care in General Practice in 20 Countries (inSIGHT): Protocol and Rationale Study. JMIR Res Protoc.

[CR45] Neves AL, Li E, Gupta PP, Fontana G, Darzi A (2021). Virtual primary care in high-income countries during the COVID-19 pandemic: Policy responses and lessons for the future. Eur J Gen Pract.

[CR46] Neves A L, van Dael J, O'Brien N, Flott K, Ghafur S, Darzi A and Mayer E (2021c). Use and impact of virtual primary care on quality and safety: The public's perspectives during the COVID-19 pandemic. J Telemed Telecare 0(0): 1357633X211066235. 10.1177/1357633X211066235. 10.1177/1357633X211066235 PMC10792996 34935535

[CR47] Office for National Statistics (2022) Which jobs can be done from home? https://www.ons.gov.uk/employmentandlabourmarket/peopleinwork/employmentandemployeetypes/articles/whichjobscanbedonefromhome/2020-07-21. Accessed 12/04/22

[CR48] Persaud N, Bedard M, Boozary A, Glazier R H, Gomes T, Hwang S W, Juni P, Law M R, Mamdani M, Manns B, Martin D, Morgan S G, Oh P, Pinto A D, Shah B R, Sullivan F, Umali N, Thorpe K E, Tu K, Laupacis A. Carefully and Easily Accessible at No Charge Medications study (2021). Adherence at 2 years with distribution of essential medicines at no charge: The CLEAN Meds randomized clinical trial. PLoS Med 18(5): e1003590. 10.1371/journal.pmed.1003590 10.1371/journal.pmed.1003590 PMC8139488 34019540

[CR49] Pharmacy Services Negotiating Committee (2020) Pandemic Delivery Service. https://psnc.org.uk/the-healthcare-landscape/covid19/pandemic-delivery-service/. Accessed 10/12/21

[CR50] Rathbone AP, Jamie K, Todd A, Husband A (2021). A qualitative study exploring the lived experience of medication use in different disease states: Linking experiences of disease symptoms to medication adherence. J Clin Pharm Ther.

[CR51] Royal College of General Practitioners (2020a) RSC workload Observatory. https://www.rcgp.org.uk/about-us/news/2020/July/rcgp-survey-provides-snapshot-of-how-gp-care-is-accessed-in-latest-stages-of-pandemic.aspx. Accessed 09/07/2022

[CR52] Royal College of General Practitioners (2020b) RCGP survey provides snapshot of how GP care is accessed in latest stages of pandemic. https://www.rcgp.org.uk/about-us/news/2020/July/rcgp-survey-provides-snapshot-of-how-gp-care-is-accessed-in-latest-stages-of-pandemic.aspx. Accessed 09/07/2022

[CR53] Sinnott SJ, Buckley C, O'Riordan D, Bradley C, Whelton H (2013). The effect of copayments for prescriptions on adherence to prescription medicines in publicly insured populations; a systematic review and meta-analysis. PLoS One.

[CR54] Stirratt MJ, Dunbar-Jacob J, Crane HM, Simoni JM, Czajkowski S, Hilliard ME, Aikens JE, Hunter CM, Velligan DI, Huntley K, Ogedegbe G, Rand CS, Schron E, Nilsen WJ (2015). Self-report measures of medication adherence behavior: recommendations on optimal use. Transl Behav Med.

[CR55] Subathra GN, Rajendrababu SR, Senthilkumar VA, Mani I, Udayakumar B (2021). Impact of COVID-19 on follow-up and medication adherence in patients with glaucoma in a tertiary eye care centre in south India. Indian J Ophthalmol.

[CR56] Trakoli A (2021). Treatment burden and ability to work. Breathe (Sheff).

[CR57] Trueman P, Lowson K, Blighe A, Meszaros A, Wright D, Glanville J, Taylor D, Newbould J, Bury M, Barber N, Jani Y (2010). Evaluation of the Scale, Causes and Costs of Waste Medicines York Health Economics Consortium and The School of Pharmacy.

[CR58] Tunceli K, Iglay K, Zhao C, Brodovicz KG, Radican L (2015). Factors associated with adherence to oral antihyperglycemic monotherapy in patients with type 2 diabetes mellitus in the United Kingdom. Diabetes Res Clin Pract.

[CR59] Vestbo J, Anderson JA, Calverley PM, Celli B, Ferguson GT, Jenkins C, Knobil K, Willits LR, Yates JC, Jones PW (2009). Adherence to inhaled therapy, mortality and hospital admission in COPD. Thorax.

[CR60] Viller F, Guillemin F, Briancon S, Moum T, Suurmeijer T, van den Heuvel W (1999). Compliance to drug treatment of patients with rheumatoid arthritis: a 3 year longitudinal study. J Rheumatol.

[CR61] Wang H, Yao F, Wang H, Wang C, Guo Z (2021). Medication Adherence and Influencing Factors Among Patients With Severe Mental Disorders in Low-Income Families During COVID-19 Outbreak. Front Psychiatry.

[CR62] Were MC, Li E, Tsopra R, Jimenez G, Serafini A, Gusso G, Lingner H, Fernandez MJ, Irving G, Petek D, Hoffman R, Lazic V, Memarian E, Koskela T, Collins C, Espitia SM, Clavería A, Nessler K, O’Neill BG, Hoedebecke K, Ungan M, Laranjo L, Ghafur S, Fontana G, Majeed A, Car J, Darzi A, Neves AL (2022). General practitioners’ perceptions of using virtual primary care during the COVID-19 pandemic: An international cross-sectional survey study. PLOS Digital Health.

