# Supplementary material for: What affected UK adults’ adherence to medicines during the COVID-19 pandemic? Cross-sectional survey in a representative sample of people with long-term conditions
Source: Z Gesundh Wiss. 2023 Jan 19:1–14. Online ahead of print. doi: 10.1007/s10389-022-01813-0 (PMC9849112; doi:10.1007/s10389-022-01813-0)
Supplement: Supplementary file 1 — (DOCX 75 kb) [file 10389_2022_1813_MOESM1_ESM.docx]

# Additional material

Additional file 1. Detailed survey methods.pdf [Reports detailed methods of the survey design]

Additional file 2. Questionnaire.pdf [Reports the questionnaire items]

Additional file 3. Detailed survey results.pdf [Reports detailed results of the survey]

## Appendix 1. Methods

### Data collection

The online survey was targeted at people taking prescribed medicines for LTCs. A pilot to identify the number of respondents reporting to take medicines for at least one LTC indicated about 500 relevant respondents per survey “wave”. We aimed to obtain about 1500 respondents with medicines for at least one LTC overall to allow representation across the key LTCs, which required data to be collected via three survey waves.

### Sampling

Kantar recruited a sample of UK residents aged 16+ from their existing database. Respondents were sampled using interlocking age and gender quotas. The relevant cohort within this sample included only respondents reporting taking at least one medicine for a LTC. Weights were provided by Kantar to ensure the sample was representative of the UK population with regards to gender, age, socioeconomic group and household size. Respondents invited to conduct the online survey were incentivised by a point system from Kantar (Lightspeed LLC). Anonymised data were provided for responses to three survey waves from Kantar. Ethical approval was obtained from University of Manchester Ethics Committee (ref: 2021-11485-19655, 22/06/2021).

### *Instrument*

The questionnaire items were developed following best practice standards and underwent a rigorous sense-check and validation process by members of the research team and a representative from Kantar. The wording of each question was reviewed and edited by our lay patient member of the team (AC). Free text options were given when possible for each question with drop-down menus. Themes covered were those expected to be relevant to medicines-taking behaviour: (1) sociodemographic information, (2) LTCs, medicines taken for LTCs, self-reported medicines adherence and reasons for non-adherence, (3) access to medicines (use and barriers to use of GPs, pharmacies, social networks, and digital and remote interventions in obtaining and taking medicines), (4) COVID-19 related behaviours and experiences. See Appendix 1 for full questionnaire.

#### Socio-demographic information

As part of each survey, Kantar collects data on sociodemographic variables (age, gender, region, household size, work status, socioeconomic group, marital status and number of children). We also asked for collection of information on ethnicity, rural/urban location and index of multiple deprivation (IMD). To identify respondents on medicines for LTCs, respondents were asked to select LTCs for which they took prescribed medicines from a dropdown menu, with the option to type in any other LTC. To differentiate between new and established LTCs, respondents were asked for each LTC if it was new since March 2020, the start of the COVID-19 pandemic.

#### Medicines and medicines adherence

For each LTC, respondents were asked to type in the name of the medicines they take for the specific LTC, if it was a new medicine, and if they were adherent. Self-reported non-adherence to medicines was defined as missing at least one dose of a medicine in the last seven days. The question was introduced with a statement that normalises non-adherence (“People often miss taking their medicines, for a whole range of reasons.”), and used a specified and short recall period for adherence behaviour as recommended for adherence measures (Stirratt, Dunbar-Jacob et al. 2015). This method for detecting non-adherence has been widely used (Clifford, Barber et al. 2006, Elliott, Boyd et al. 2020, Persaud, Bedard et al. 2021) and was found to report similar results compared with the more complex and widely accepted Morisky Medication Adherence Scale (MMAS) (Elliott, Boyd et al. 2020).

We used the capabilities, opportunities and motivations model of behaviour change (COM-B) (Keyworth, Epton et al. 2020), a validated theoretical framework endorsed by the UK National Institute for Health and Care Excellence (National Institute for Health and Care Excellence 2014) to understand reasons for non-adherence. Respondents were asked to report by how much they agreed (strongly disagree [0] to strongly agree [10]) with each of the 6 items describing potential reasons for non-adherence: physical capability, psychological capability, physical opportunity, social opportunity, reflective motivation, and automatic motivation(Keyworth, Epton et al. 2020). Each of these items was described and examples were given to ensure respondents understood their meaning [Appendix 1].

#### Access to medicines

Respondents were asked about access to general practitioner (GP) or community pharmacy services: how prescriptions and medicines were accessed, times with no access to medicine, reasons for access problems, and actions taken to resolve access problems. Drop-down menus were reviewed and edited by the patient representative and the research team.

The questionnaire included items around general factors affecting medicines access (prescription fees, prepayment certificates, car ownership, availability of public transport, help with taking medicines at home, and uptake of digital tools to support medicines management) and COVID-19 related factors (shielding status, COVID-19 infection, COVID-19 treatment, and COVID-19 avoidance behaviour). The drop down menu for potential COVID-19 avoidance behaviours was based on the Opinions and Lifestyle Survey from the Office for National Statistics on the impact of the COVID-19 pandemic (Taylor, Ainslie et al. 2021).

### Data preparation

Only adults who reported taking a medicine for an LTC were included in our final cohort for analysis. Respondents reported medicines they took by typing in the medicine by hand. These medicines were grouped according to British National Formulary (BNF) therapeutic groups (National Institute for Health and Care Excellence 2019).

### Statistical analysis

Descriptive statistics were used to characterise the population and report results around the themes of medicines, medicines adherence and factors affecting access. Adherence was expressed as binary variable, where a respondent was considered non-adherent to a medicine if they reported missing at least one dose of a medicine in the last seven days.

Reasons for non-adherence according to the COM-B were described descriptively to illustrate perceptions of capabilities, opportunities and motivations. One-way repeated measures (within-participant) ANOVA or the Friedman test were used to explore difference between levels of capabilities, opportunities and motivations with respect to adhering to their medicines. The non-parametric Friedman test was used if the assumption of normality required to perform a repeated measures ANOVA was not met.

Logistic regression analyses were used to model associations between these factors and medicines adherence. For the logistic regression analysis, survey weights provided by Kantar ensured the analysis appropriately reflected the UK population. In univariate logistic regression analysis, we investigated statistically significant (p<0.05) associations with adherence of: (1) sociodemographic factors (age, ethnicity, region, rural/urban location, work status, socio-economic group, household size, IMD), (2) number of medicines taken, (3) medicine types by therapeutic group, (4) newly diagnosed LTCs or prescribed medicines, (5) Covid-related factors (shielding status and positive COVID-19 test), any avoidance behaviour~~,~~ (6) factors associated with access to medicine (car ownership, help taking medicines at home, exempt from prescription fee), (7) access problems (no access to medicine, emergency supply uptake, substitution with non-prescription medicine), and (8) use of digital tools to order medicines or as a reminder to take medicines. We did not include the ownership of a prepayment certificate because missingness was extremely high (73.3%). Information on reasons for access problems and actions taken to resolve them, as well as COVID-infection treatment details were not included in the logistic regression analysis because patient numbers for each response option were small and these questions were only answered by a subset of the population, those with access problems and a positive COVID-19 infection, respectively. Hence, missingness for these variables were by design almost 90% and 80% respectively. Respondents were identified as users of digital tools if they reported to have used the NHS app or a GP app the last time they ordered their prescription [question 15] or reported to use digital tools to support them to take their medicine [question 26].

In multiple logistic regression, using adherence as the dependent variable identified associations that were found significant in the univariate analysis which were then tested adjusting for potential explanatory factors. All variables significant in univariate analysis were adjusted for. The type of LTCs were not considered in the analysis because we assumed that the impact of the LTC would be represented by the type of medicine prescribed for it.

All analysis was conducted using *Stata MP 16 (StataCorp.. Stata Statistical Software: Release 16. College Station 2019)*. We used linktest to test for model specification errors (Pregibon 1979) and the variance inflation factor to test for multicollinearity. General goodness of fit was tested using the *Stata* command svylogitgof that performs the F-adjusted mean residual test and that can be used in weighted survey analysis in contrast to other commonly used goodness of fit commands in *Stata MP 16*  (Archer, Lemeshow et al. 2007).

## Appendix 2. Questionnaire

*Themes:*

Number/type of new/established LTCs and prescription medicines

COVID-19/shielding status, rural/urban, prescription exemption status, car ownership

COVID-19-avoidance behaviours

COVID-19 infections, hospital admissions

Access to/use of GP/community pharmacy and use of digital tools to obtain prescriptions

Use of non-prescription medicines

Medicines adherence, reasons for adherence, use of digital tools to increase adherence

**Number/type of new/established LTCs and prescription medicines**

| Q No. | Category | Question text | Answer format |
| --- | --- | --- | --- |
| A1 | Filter question | Are you taking prescription medication for any of the following conditions? Please select all that apply | Drop-down menu |
| A2 | Medicines for each disease details | You have said that you take medicines for <<insert disease selected>>. What are the names of these medicines? | Free-text, one box for each condition, patient can free-type in all meds |
| A3 | New/Est LTC | Have any of the conditions below <<insert all diseases selected>> been newly diagnosed since March 2020? | Yes/no for each disease |
| A4 | New/Est medicines | You have told us you take the medicines listed below: <<list responses to Q2>> Have any of these medicines been newly prescribed since March 2020? | Yes/no for each medicine |

**COVID-19 shielding status, rural/urban, prescription exemption status, car ownership**

| Q No. | Category | Question text | Answer format |
| --- | --- | --- | --- |
| B1 | Shielding status | During the COVID pandemic, some people have been identified as clinically extremely vulnerable. If you are in this group, you will previously have received a letter from the NHS or from your GP telling you this. You may have been advised to shield in the past. Have you been told to shield at any point since March 2020? | Yes/no |
| B2 | Rx exemption status | Are you exempt from paying prescription charges? (ONLY ASK IN ENGLAND) | Yes/No |
| B3 | Prepayment certificate | Do you have a prepayment certificate to help with the costs of your prescription medicines? (ONLY ASK IN ENGLAND) | Yes/No |
| B4 | Car ownership | Do you own/have access to a car? | Yes/No |
| B5 | Access to public transport | Are there public transport options in your area suitable for your needs? | Yes/No |
| B6 | Rural/urban and IMD | >> will be mapped from post codes by Kantar |  |
| B9 | Carer support 2 | Does someone else in your household (e.g., wife, husband) or a carer help you take your prescription medicines? | Yes/No |
| B10 | Living alone | >> Household size is already collected by Kantar | Yes/No |

**COVID-19-avoidance behaviours**

| Q No. | Category | Question text | Answer format |
| --- | --- | --- | --- |
| C1 | COVID behaviour | In the last 7 days how often have you done the following to keep you safe from COVID? | Drop-down menu |

**COVID-19 infections, hospital admissions**

| Q No. | Category | Question text | Answer format |
| --- | --- | --- | --- |
| D1 | COVID status | Have you tested positive for COVID since March 2020? | Yes/no |
| D2 | COVID infection | Have you been treated for a COVID infection since March 2020? | Yes/no |
| D3 | COVID hospital stay | Have you had to stay overnight in hospital for a COVID infection since March 2020? | Yes/no |

**Access to/use of GP/community pharmacy**

| Q No. | Category | Question text | Answer format |
| --- | --- | --- | --- |
| E1 | Prescription ordering | How did you order your prescription at your GP the last time you renewed your prescriptions? | Drop-down menu |
| E2 | Medicine collection | How did you collect or receive your medicines the last time you renewed your prescriptions? | Drop-down menu |
| E3 | Access to GP/pharmacy | Have you had any problem obtaining/filling a prescription for any of your medicines in the last two months? | Yes/no for each medicine |
| E4 | Access to GP/ pharmacy | Which of the following reasons account for this problem? <<tick all that apply>> | Drop-down menu |
| E5 | Access to GP | Which of the following actions have you taken to get the prescription from your GP? <<tick all that apply>> | Drop-down menu |
| E6 | Access to pharmacy | Which of the following actions have you taken to fill your prescription at a pharmacy? <<tick all that apply>> | Drop-down menu |

**Use of non-prescription medicines**

| Q No. | Category | Question text | Answer format |
| --- | --- | --- | --- |
| F1 | Use of OTC medicines | Since March 2020, how many times have you bought non-prescription medicines from a pharmacy, a shop, or online, **instead of getting a prescription medicine** (this might be antihistamines, painkillers, anti-inflammatories, medicines for heartburn or indigestion, alternative/herbal remedy)? | Never, yes (Scale from “0” to “7 or more”) |
| F2 | Emergency supply | Since March 2020, how often have you run out of your medicine and had to obtain an immediate supply from you pharmacist until your next prescription? | Never, yes (Scale from “0” to “7 or more”) |

**Medicines adherence, reasons for non-adherence**

| Q No. | Category | Question text | Answer format |
| --- | --- | --- | --- |
| G1 | Adherence | You have told us that you take the following medicines:  <<generate list of medicines>>  People often miss taking their medicines, for a whole range of reasons. For each of your medicines, we would like you to tell us how many doses have you missed in the last 7 days? | Scale from “0” to “7 or more” |
| G2-G7: only ask if G1 is >0 | | | |
| G2 | Reasons for non-adherence | I missed taking my medicine because I did not have the **physical opportunity** to take my medicines as prescribed  Explanation box: What is PHYSICAL opportunity?  The environment provides the opportunity to engage in the activity concerned. (e.g., I did not have time, the things I needed, I could not get the prescription from my GP or pharmacy) | Scale [strongly disagree (0) to strongly agree (10)] |
| G3 | Reasons for non-adherence | I missed taking my medicine because I did not have the **social opportunity** to take my medicines as prescribed  Explanation box: What is SOCIAL opportunity?  Interpersonal influences, social cues and cultural norms provide the opportunity to engage in the activity concerned (e.g., lack of support from family or friends, fasting during lent/Ramadan) | Scale [strongly disagree (0) to strongly agree (10)] |
| G4 | Reasons for non-adherence | I missed taking my medicine because I was not **motivated** to take my medicines as prescribed  Explanation box: What is motivation?  Conscious planning (beliefs about what is good and bad). (e.g., I did not feel the need to, I am worried I feel worse when I take it, it does not work, my condition is under control) | Scale [strongly disagree (0) to strongly agree (10)] |
| G5 | Reasons for non-adherence | I just missed taking my medicine as prescribed **automatically**  Explanation box: Automatic motivation involves doing something without thinking or having to consciously remember (e.g. ‘is something I did accidentally’, I just forgot) | Scale [strongly disagree (0) to strongly agree (10)] |
| G6 | Reasons for non-adherence | I missed taking my medicine because I was not **physically** able to take my medicines as prescribed  Explanation box: What is physical capability?  Having the physical skill, strength or stamina to take my medicine  (e.g., my arthritis won’t allow me to open the bottle, I could not see which medicines I opened, I struggle to swallow my medicine) | Scale [strongly disagree (0) to strongly agree (10)] |
| G7 | Reasons for non-adherence | I missed taking my medicine because I was not **psychologically** able to take my medicines as prescribed  Explanation box: What is psychological capability?  Knowledge and/or psychological skills  (e.g.,I don’t remember if I took my medicine already, don’t remember to take my medicines with me when I leave the house, I don’t know how to take them). | Scale [strongly disagree (0) to strongly agree (10)] |
| G8 | Any other information | Is there any other information or experiences you would like to share with us about obtaining or taking prescription medicines during the COVID pandemic? | Free-text |
| G9 | Digital tools | Did you use any apps or digital tools to support you to take your medicine? <<tick all that apply>> | Drop-down menu |

Drop-down menus

APPENDIX: Drop-down menu for diseases A1

| Do you have any long-term conditions |
| --- |
| Type 1 diabetes |
| Type 2 diabetes |
| Asthma |
| Chronic obstructive airways disease (COPD) |
| Sleep apnoea |
| Arthritis (osteoarthritis, rheumatoid arthritis, ankylosing spondylitis) |
| Gout |
| Lupus |
| Arrhythmia/atrial fibrillation |
| High blood pressure |
| Heart disease/angina |
| Heart failure |
| Peripheral vascular disease |
| History of a heart attack or stroke |
| Chronic pain |
| Osteoporosis |
| Fibromyalgia/chronic widespread pain |
| Back pain |
| Migraine |
| GI disorder (ulcer, heartburn) |
| Inflammatory bowel disease |
| Crohn’s disease |
| Thyroid/metabolic disorder (thyrotoxicosis, hypothyroidism, hypogonadism, Cushing syndrome, Addison’s disease) |
| Chronic kidney disease |
| Glaucoma |
| Cancer |
| HIV |
| Depression |
| Anxiety |
| Bipolar disorder |
| Schizophrenia |
| Epilepsy |
| Parkinson’s Disease |
| Eczema |
| Psoriasis |
| Any other long term condition, namely... |
| None of the above |
| Prefer not to say |

Source: https://www.ncbi.nlm.nih.gov/books/NBK263824/

APPENDIX: Drop-down for COVID avoidance measures C1

| In the last 7 days how often have you done the following to keep you safe from COVID?  Always/often washed my hands with soap and water after returning home from a public place  Used a face covering when inside public places or on public transport  Used a face covering while walking or exercising outdoors  Always/often maintained social distancing when meeting up with people outside your support bubble  Avoided physical contact when outside my home  Avoided contact with older people or other vulnerable people because of the Coronavirus (COVID-19) pandemic?  Self-isolated when I had symptoms  Stayed at home or only left for work, physical activity, essential shopping or medical needs.  Did not allow people outside my bubble into my home  Worked from home because of the Coronavirus (COVID-19) pandemic? |
| --- |

Source: From COVID-19 Infection Survey – Office for National Statistics: Coronavirus and the social impact on Great Britain [https://www.ons.gov.uk/peoplepopulationandcommunity/healthandsocialcare/healthandwellbeing/datasets/coronavirusandthesocialimpactsongreatbritaindata];

APPENDIX: drop-down for ways to order prescription at GP (status quo) E1

| How did you order your prescription at your GP the last time you renewed your prescriptions?  I ordered my prescription in person at a GP visit/appointment  Friend, relative or volunteer ordered prescription for me  I ordered my prescription at my GP by email  I ordered my prescription from my GP by app  I ordered my prescription from my GP by telephone  I ordered my prescription online on my GPs website  I ordered my prescription via a website (e.g., patient.org)  I ordered my prescription on the NHS app  Local action/support group ordered it for me (e.g., through Facebook, churches, neighbours)  I do not collect or receive my medicines at the moment  Free text |
| --- |

APPENDIX: drop-down for ways to collect medicine at pharmacy (status quo) E2

| How did you collect or receive your medicines the last time you renewed your prescriptions?  Collect medicines by yourself at the pharmacy  Medicines are picked up by friends or family at the pharmacy  Medicines are picked up by a formal carer at the pharmacy  Medicines are delivered by the local pharmacy  Medicines are delivered through/by a local action/support group (e.g., through Facebook group, churches, neighbours)  Medicines are delivered by post by an online prescription service (e.g., pharmacy2you, pill time, PillPack, CareZone …)  I do not collect or receive my medicines at the moment  Free text |
| --- |

APPENDIX: Drop-down for Reasons for not obtaining prescriptions (GP/pharmacy access) E4

| I had to self-isolate  I did not want to leave the house  I was not able to order a prescription by telephone  I was not able to order a prescription by email/app  I did not want to use public transport  I wasn’t allowed to go into the GP practice  I don’t think my medicine is very important and did not want to bother my GP  The GP practice did not allow to order by phone  Only online orders available at my GP and I don’t have internet access  Only online orders available at my GP and I do not know how to order online  Only online orders available at my GP and I do not want to use them  I did not get an appointment at my GP practice  Pharmacy was closed  Queue was too long at the pharmacy  Pharmacy did not do home deliveries  The weather was bad  People weren’t social distancing in the queue, and I did not feel safe  The pharmacy was too far away  Medicines were out of stock or unavailable  Free text |
| --- |

APPENDIX: Drop-down for actions to get round this problem (GP access) E5

| Which of the following actions have you taken to get the prescription from your GP?  I missed my prescription  Friend, relative or volunteer ordered prescription for me  I did it reluctantly but I was very concerned  I ordered my prescription at my GP by email  I ordered my prescription from my GP by app  I ordered my prescription from my GP by telephone  I ordered my prescription online on my GPs website  Local action/support group ordered it for me (e.g., through Facebook, churches, neighbours)  I broke my isolation because I had no choice  I accessed A+E  I accessed an out of hours GP service  I visited a walk-in centre  I requested an emergency supply from the pharmacy  I accessed an NHS helpline  Free text |
| --- |

APPENDIX: Drop-down for actions to get around this problem (pharmacy actions) E6

| Which of the following actions have you taken to fill your prescription at a pharmacy?  Friend, relative or volunteer collected prescription  I had to break my self-isolation  I did it reluctantly but I was very concerned  I used an online prescription service (e.g., pharmacy2you, pill time, PillPack, CareZone)  Local action/support group got it for me (e.g., through Facebook group, churches, neighbours)  My pharmacy agreed to deliver my medicine  I used a different pharmacy  Free text |
| --- |

Source: drop-down menus for E1-E6 were reviewed and edited with the patient representative Antony Chuter and externally by representatives from the research team from Boots

Appendix: Drop-down for uptake of digital tools to support medicines taking G9

| Did you use any apps or digital tools to support you to take your medicine? |
| --- |
| No, I don’t use apps or digital tools  Yes, I let a digital home assistant remind me to take my medicine (e.g., Alexa or google)  Yes, I use a dispenser that sends me alerts when to take my medicine (e.g. MedMinder)  Yes, I use an app that reminds me to renew my prescription  Yes, I use an app to remind me to take my medicines (e.g.,Medisafe, CareZone, Mango Health, bedsider reminders, MyTherapy, Pill Reminder All in One)  Yes I use a handheld reminder device (e.g., TabTime, e-pill time cap)  Yes, I use an online pharmacy that delivers dosed out medications in separate packages (e.g., PillPack, CareZone)  Yes, I use an online pharmacy service that handles ordering my repeat prescription for me (e.g., PillPack, CareZone)  Yes, I set reminders on my phone  Yes, I use <<free text>> |

## Appendix 3. Detailed survey results

This appendix provides detailed survey responses on (Table 1) respondent characteristics, (Table 2) how prescriptions were ordered at the GP, (Table 3) how prescriptions were filled from the pharmacy, (Table 4) how people deal with access issues, (Table 5) adherence, (Table 6) factors affecting adherence, and (Table 7) results of the logistic regression analysis .

| Table 1. Respondent characteristics (N=1746) |  |
| --- | --- |
| Sociodemographic characteristics | Number of respondents (%) |
| Age group |  |
| 16-24 | 157 (9.0%) |
| 25-34 | 221 (12.7%) |
| 35-44 | 234 (13.4%) |
| 45-54 | 331 (19.0%) |
| 55-64 | 319 (18.3%) |
| 65 or older | 484 (27.7%) |
| Gender |  |
| Male | 794 (45.5%) |
| Female | 949 (54.4%) |
| Unknown | 3 (0.2%) |
| Ethnicity |  |
| White | 1,529 (87.6%) |
| BAME | 148 (8.5%) |
| Missing | 69 (4.0%) |
| Region |  |
| Scotland | 170 (9.7%) |
| North East | 56 (3.2%) |
| Yorkshire/Humber | 140 (8.0%) |
| North West | 234 (13.4%) |
| East Midlands | 130 (7.4%) |
| West Midlands | 123 (7.0%) |
| South East | 228 (13.1%) |
| East of England | 203 (11.6%) |
| Greater London | 171 (9.8%) |
| Wales | 162 (9.3%) |
| West | 98 (5.6%) |
| Northern Ireland | 31 (1.8%) |
| Long term condition (LTC) |  |
| Any GI condition | 135 (7.7%) |
| GI disorder (ulcer, heartburn) | 73 (4.2%) |
| Inflammatory bowel disease | 50 (2.9%) |
| Crohn’s disease | 25 (1.4%) |
| Any CV condition | 581 (33.3%) |
| High blood pressure | 502 (28.8%) |
| Heart disease/angina | 49 (2.8%) |
| Heart failure | 33 (1.9%) |
| Peripheral vascular disease | 6 (0.3%) |
| History of a heart attack or stroke | 60 (3.4%) |
| High cholesterol | 8 (11.8%) |
| Arrhythmia/atrial fibrillation | 45 (2.6%) |
| Any respiratory condition | 362 (20.7%) |
| Asthma | 292 (16.7%) |
| Chronic obstructive airways disease (COPD) | 55 (3.2%) |
| Sleep apnoea | 38 (2.2%) |
| Any CNS condition | 942 (54.0%) |
| Any mental health condition | 585 (33.5%) |
| Depression | 417 (23.9%) |
| Anxiety | 393 (22.5%) |
| Bipolar disorder | 32 (1.8%) |
| Schizophrenia | 15 (0.9%) |
| Other mental health condition | 7 (0.4%) |
| Fibromyalgia/chronic widespread pain | 67 (3.8%) |
| Back pain | 247 (14.1%) |
| Migraine | 185 (10.6%) |
| Chronic pain | 160 (9.2%) |
| Epilepsy | 45 (2.6%) |
| Multiple sclerosis | 5 (0.3%) |
| HIV infectious | 13 (0.7%) |
| Any endocrine condition | 414 (23.7%) |
| Type 1 diabetes | 42 (2.4%) |
| Type 2 diabetes | 213 (12.2%) |
| Thyroid/metabolic disorder | 125 (7.2%) |
| Chronic kidney disease | 12 (0.7%) |
| Osteoporosis | 46 (2.6%) |
| Any malignant disease | 29 (1.7%) |
| Cancer | 29 (1.7%) |
| Any rheumatic condition | 251 (14.4%) |
| Arthritis (osteoarthritis, rheumatoid arthritis, ankylosing spondylitis) | 194 (11.1%) |
| Gout | 57 (3.3%) |
| Lupus erythematosus | 12 (0.7%) |
| Opthalmic condition | 35 (2.0%) |
| Glaucoma | 35 (2.0%) |
| Any skin condition | 159 (9.1%) |
| Eczema | 115 (6.6%) |
| Psoriasis | 54 (3.1%) |
| At least on unknown condition ^a^ | 48 (2.7%) |
| Type of medicine |  |
| GI-system medicine | 121 (6.9%) |
| At least one cardiovascular medicine | 605 (34.7%) |
| Positive inotropic medicines (cardiac glycoside) | 9 (0.5%) |
| Diuretics | 61 (3.5%) |
| Beta-blocker | 180 (10.3%) |
| Hypertension and heart failure medicines (alpha-blocker and RAS medicines) | 331 (19.0%) |
| Nitrates, calcium-channel blockers & other antianginal medicines | 193 (11.1%) |
| Oral anticoagulants | 29 (1.7%) |
| Antiplatelets | 70 (4.0%) |
| Lipid regulating medicines (statins) | 86 (4.9%) |
| Antiarrhythmic medicines | 4 (0.2%) |
| At least one respiratory medicine | 309 (17.7%) |
| Bronchodilator (LABA/SABA/other) | 201 (11.5%) |
| Inhaled corticosteroid | 79 (4.5%) |
| Combination inhaler corticosteroid/LABA | 82 (4.7%) |
| Unspecified inhaler | 41 (2.3%) |
| Antihistamines | 21 (1.2%) |
| Other respiratory medicines, oral application | 20 (1.1%) |
| At least one CNS medicine | 923 (52.9%) |
| Hypnotics and anxiolytics | 50 (2.9%) |
| Antipsychotics | 37 (2.1%) |
| Antidepressants | 512 (29.3%) |
| Analgesics used for migraine | 155 (8.9%) |
| Other analgesics | 359 (20.6%) |
| Antiepileptics | 82 (4.7%) |
| Drugs used in nausea and vertigo | 2 (0.1%) |
| Drugs used in parkinsonism and related disorders | 4 (0.2%) |
| At least one infection medicine (antibiotics/HIV) | 5 (0.3%) |
| At least one endocrine system medicine | 351 (20.1%) |
| Oral antidiabetics | 65 (3.7%) |
| Insulin | 186 (10.7%) |
| Thyroid and antithyroid medicines | 116 (6.6%) |
| Corticosteroids | 12 (0.7%) |
| Drugs affecting bone metabolism | 13 (0.7%) |
| At least one urinary tract disorder medicine | 2 (0.1%) |
| At least one malignant disease or immunosuppression medicine | 33 (1.9%) |
| At least one medicine for nutrition and blood including herbal remedies | 26 (1.5%) |
| At least one medicine used in rheumatic disease and gout | 66 (3.8%) |
| At least one type of eye drops | 23 (1.3%) |
| At least one topical preparation | 84 (4.8%) |
| Topical corticosteroids | 36 (2.1%) |
| Other topical preparation (emollient/eczema) | 59 (3.4%) |
| Corticosteroid with unspecified application | 35 (2.0%) |
| At least one unknown medicine ^a^ | 377 (21.6%) |
| Data are presented as n (%). ^a^ specified as other, unknown or unrecognisable |  |

| Table 2. Access to medicines - ordering prescriptions from the general practice (N=1746) | |
| --- | --- |
| Medication ordering process at GP ^a^ | Number of respondents (%) |
| I ordered my prescription in person at a GP visit/appointment | 124 (7.1%) |
| Friend, relative or volunteer ordered my prescription for me | 39 (2.2%) |
| I ordered my prescription at my GP by email | 77 (4.4%) |
| I ordered my prescription from my GP by app | 149 (8.5%) |
| I ordered my prescription from my GP by telephone | 248 (14.2%) |
| I ordered my prescription online on my GP's website | 366 (21.0%) |
| I ordered my prescription via a website (e.g. patient.org) | 224 (12.8%) |
| I ordered my prescription on the NHS app | 98 (5.6%) |
| Local action/support group ordered it for me (e.g. through Facebook, churches, neighbours) | 13 (0.7%) |
| I ordered my prescription from my designated pharmacy | 357 (20.4%) |
| I do not collect or receive my medicines at the moment | 37 (2.1%) |
| Other | 6 (0.3%) |
| Drop off request in box at doctor | 8 (0.5%) |
| Data are presented as n (%). ^a^ Based on the last time the respondent ordered medication |  |

| Table 3. Access to medicines - filling prescriptions from the pharmacy (N=1746) | |
| --- | --- |
| Medication collection process at pharmacy ^a^ | Number of respondents (%) |
| Collected medicines by yourself at the pharmacy | 1,048 (60.0%) |
| Medicines were picked up by friends or family at the pharmacy | 245 (14.0%) |
| Medicines were picked up by a formal carer at the pharmacy | 37 (2.1%) |
| Medicines were delivered by the local pharmacy | 223 (12.8%) |
| Medicines were delivered through/by a local action/support group (e.g., through Facebook group, churches, neighbours) | 37 (2.1%) |
| Medicines were delivered by post by an online prescription service (e.g., pharmacy2you, pill time, PillPack, CareZone …) | 130 (7.4%) |
| I do not collect or receive my medicines at the moment | 21 (1.2%) |
| Other | 5 (0.3%) |
| Data are presented as n (%). ^a^ Based on the last time the respondent filled their prescription |  |

| Table 4. Actions taken to resolve access problems (N=182) |  |
| --- | --- |
| Actions taken to resolve problems with GP access | Number of respondents (%) |
| I missed my prescription | 23 (12.6%) |
| Friend, relative or volunteer ordered prescription for me | 24 (13.2%) |
| I ordered my prescription at my GP by email | 12 (6.6%) |
| I ordered my prescription from my GP by app | 15 (8.2%) |
| I ordered my prescription from my GP by telephone | 27 (14.8%) |
| I ordered my prescription online on my GPs website | 26 (14.3%) |
| Local action/support group ordered it for me (e.g., through Facebook, churches, neighbours)* | 16 (8.8%) |
| I broke my isolation because I had no choice | 9 (4.9%) |
| I accessed A+E | 16 (8.8%) |
| I accessed an out of hours GP service | 17 (9.3%) |
| I visited a walk-in centre | 14 (7.7%) |
| I requested an emergency supply from the pharmacy | 22 (12.1%) |
| I accessed an NHS helpline | 18 (9.9%) |
| Other | 3 (1.6%) |
| The problem I experienced was not related to the GP | 15 (8.2%) |
| No action taken to resolve the problem | 1 (0.5%) |
| Actions taken to resolve problem with filling prescription at the pharmacy |  |
| Friend, relative or volunteer collected prescription | 41 (22.5%) |
| I had to break my self-isolation | 20 (11.0%) |
| I used an online prescription service (e.g., pharmacy2you, pill time, PillPack, CareZone …) | 32 (17.6%) |
| Local action/support group ordered it for me (e.g., through Facebook, churches, neighbours) | 39 (21.4%) |
| My pharmacy agreed to deliver my medicine | 32 (17.6%) |
| I used a different pharmacy | 37 (20.3%) |
| Other | 4 (2.2%) |
| The problem I experienced was not related to the pharmacy | 30 (16.5%) |
| None | 2 (1.1%) |
|  |  |

| Table 5. Non-adherence by prescribed medicine | | |
| --- | --- | --- |
| Type of medicine | Number of respondents (n)^a^ | Nonadherence (%)^b^ |
| Any medicine | 1746 | 394 (22.6%) |
| GI-system medicine | 121 | 24 (19.8%) |
| Cardiovascular medicine | 605 | 97 (16.0%) |
| Positive inotropic medicines (cardiac glycosides) | 9 | 0 (0.0%) |
| Diuretics | 61 | 6 (9.8%) |
| Beta-blocking medicines | 180 | 32 (17.8%) |
| Hypertension/heart failure medicines (alpha-blocker and RAS medicines) | 331 | 44 (13.3%) |
| Nitrates, calcium-channel blockers & other antianginal medicines | 193 | 22 (11.4%) |
| Oral anticoagulants | 29 | 4 (13.8%) |
| Antiplatelet medicines | 70 | 16 (22.9%) |
| Lipid regulating medicines | 86 | 23 (26.7%) |
| Antiarrhythmic medicines | 4 | 0 (0.0%) |
| Respiratory medicines | 309 | 75 (24.3%) |
| Bronchodilator (LABA/SABA/other) | 201 | 42 (20.9%) |
| Inhaled corticosteroids | 79 | 15 (19.0%) |
| Combination inhaler corticosteroid/LABA | 82 | 16 (19.5%) |
| Unspecified inhaler | 41 | 10 (24.4%) |
| Antihistamines | 21 | 11 (52.4%) |
| Other respiratory medicines, oral application | 20 | 5 (25.0%) |
| CNS medicines | 923 | 284 (30.8%) |
| Hypnotics and anxiolytics | 50 | 14 (28.0%) |
| Antipsychotics | 37 | 8 (21.6%) |
| Antidepressants | 512 | 140 (27.3%) |
| Analgesics | 514 | 162 (31.5%) |
| Antiepileptics | 82 | 10 (12.2%) |
| Drugs used in nausea and vertigo | 2 | 2 (100.0%) |
| Drugs used in parkinsonism and related disorders | 4 | 2 (50.0%) |
| Infection medicine | 5 | 3 (60.0%) |
| Endocrine system medicines | 351 | 79 (22.5%) |
| Diabetes (insulin and oral antidiabetics) | 228 | 58 (25.4%) |
| Thyroid and antithyroid medicines | 116 | 19 (16.4%) |
| Corticosteroids | 12 | 3 (25.0%) |
| Drugs affecting bone metabolism | 13 | 3 (23.1%) |
| Urinary tract disorder medicine | 2 | 0 (0.0%) |
| Malignant disease or immunosuppression medicine | 33 | 9 (27.3%) |
| Nutrition and blood including herbal remedies | 26 | 6 (23.1%) |
| Drugs used in rheumatic disease and gout | 66 | 13 (19.7%) |
| Eye drops | 23 | 2 (8.7%) |
| Topical preparations | 84 | 30 (35.7%) |
| Topical corticosteroids | 36 | 11 (30.6%) |
| Other topical preparations (emollient/eczema) | 59 | 25 (42.4%) |
| Corticosteroid with unspecified application | 35 | 7 (20.0%) |
| Unspecified medicine | 377 | 115 (30.5%) |
| Data are presented as n (%). ^a^ Respondents reporting at least one medicine in this medicine group; ^b^ Respondents reporting nonadherence to at least one medicine in this medicine group | | |

| Table 5. Summary measures and reasons for non-adherence (N=394) | |
| --- | --- |
| Measure of non-adherence | Mean (SD) |
| Total doses missed in last 7 days | 6.1 (8.3) |
| Mean number of missed doses per medicine in last 7 days | 3.0 (2.0) |
| Number of medicines with missed doses in last 7 days | 2.0 (1.8) |
| Reasons for non-adherence | Mean score (SD) |
| Physical opportunity | 5.3 (3.5) |
| Social opportunity | 5.1 (3.5) |
| Reflective motivation | 5.7 (3.4) |
| Automatic motivation | 7.0 (3.3) |
| Physical capability | 4.9 (3.5) |
| Psychological capability | 5.4 (3.5) |
| One-Way repeated measures ANOVA |  |
| Shapiro-Wilk test | p<0.001^1^ |
| Mauchly’s test of sphericity | p<0.001^2^ |
| Friedman test | Q=130.81, p<0.001 |
| Data are presented as mean (SD). ^1^The Shapiro-Wilk test showed a p-value <0.001 for all reasons for non-adherence, indicating that normality assumption was violated; ^2^Mauchly’s test of sphericity indicated that the assumption of sphericity was violated | |

| Table 6. Factors affecting adherence (N=1746) |  |
| --- | --- |
| Variable | Number of respondents (%) |
| **General factors** |  |
| Exempt from prescription fee? |  |
| No | 467 (26.7%) |
| Yes | 980 (56.1%) |
| Missing | 299 (17.1%) |
| Prepayment certificate for prescriptions? |  |
| No | 311 (17.8%) |
| Access to a car? |  |
| No | 441 (25.3%) |
| Yes | 1,305 (74.7%) |
| Suitable public transport available? |  |
| No | 451 (25.8%) |
| Yes | 1,295 (74.2%) |
| Help taking medicines at home? |  |
| No | 1,473 (84.4%) |
| Yes | 273 (15.6%) |
| **COVID-19 related factors** |  |
| Shielded |  |
| No | 1,361 (77.9%) |
| Yes | 385 (22.1%) |
| Tested positive for Covid-19 (n=1746) |  |
| Yes | 152 (8.7%) |
| No | 1,594 (91.3%) |
| Treated for Covid-19 (n=152) |  |
| No | 62 (3.6%) |
| Yes | 90 (5.2%) |
| Missing | 1,594 (91.3%) |
| Hospitalised for Covid-19 (n=90) |  |
| No | 38 (2.2%) |
| Yes | 52 (3.0%) |
| Missing | 1,656 (94.8%) |
| Treatment for Covid-19 (n=1745) |  |
| Not infected | 1,594 (91.3%) |
| Infected, not treated | 62 (3.6%) |
| Infected, treated outside hospital | 38 (2.2%) |
| Infected, treated in hospital | 52 (3.0%) |
| Data are presented as n (%). |  |

| Table 7: Results of logistic regression analysis showing factors associated with non-adherence (N=1746) | | | |
| --- | --- | --- | --- |
|  | Model 1 | Model 2 |  |
| F-adjusted mean residual test | 0.86 | 0.99 |  |
| Independent variables | Adjusted OR (95 % CI) ^a^ | Adjusted OR (95 % CI) ^a^ |  |
| Age |  |  |  |
| 16-24 | 1 | 1 |  |
| 25-34 | 0.66 [0.36,1.22] | 0.64 [0.35,1.19] |  |
| **35-44** | **0.42 [0.22,0.79]** | **0.39 [0.21,0.73]** |  |
| **45-54** | **0.42 [0.23,0.76]** | **0.39 [0.22,0.71]** |  |
| **55-64** | **0.16 [0.08,0.32]** | **0.15 [0.07,0.30]** |  |
| **65+** | **0.15 [0.07,0.33]** | **0.14 [0.07,0.31]** |  |
| Gender |  |  |  |
| Male | 1 | 1 |  |
| **Female** | **0.60 [0.42,0.84]** | **0.60 [0.43,0.85]** |  |
| Unknown | 1.89 [0.85,4.17] | 1.93 [0.88,4.25] |  |
| Ethnicity |  |  |  |
| White | 1 | 1 |  |
| BAME | 1.08 [0.67,1.75] | 1.14 [0.71,1.82] |  |
| Unknown | 1.53 [0.75,3.14] | 1.61 [0.79,3.30] |  |
| Region |  |  |  |
| Scotland | 1.89 [0.85,4.17] | 1.93 [0.88,4.25] |  |
| North East | 1.01 [0.41,2.49] | 1.10 [0.46,2.64] |  |
| Yorkshire/Humber | 1.60 [0.74,3.46] | 1.58 [0.74,3.40] |  |
| North West | 1.75 [0.90,3.39] | 1.75 [0.91,3.36] |  |
| East Midlands | 1.18 [0.53,2.61] | 1.16 [0.53,2.54] |  |
| West Midlands | 0.86 [0.38,1.93] | 0.86 [0.39,1.94] |  |
| South East | 1.42 [0.67,3.01] | 1.39 [0.66,2.91] |  |
| East of England | 1.50 [0.72,3.13] | 1.52 [0.73,3.13] |  |
| Greater London | 1.47 [0.70,3.09] | 1.48 [0.71,3.08] |  |
| Wales | 1 | 1 |  |
| West | 1.36 [0.58,3.18] | 1.35 [0.58,3.14] |  |
| Northern Ireland | 2.25 [0.77,6.58] | 2.28 [0.79,6.60] |  |
| Socioeconomic group ^b^ |  |  |  |
| 1 | 1 | 1 |  |
| 2 | 1.41 [0.77,2.59] | 1.37 [0.75,2.49] |  |
| 3 | 1.30 [0.75,2.24] | 1.26 [0.73,2.16] |  |
| 4 | 1.50 [0.86,2.61] | 1.43 [0.83,2.47] |  |
| **5** | **2.60 [1.26,5.38]** | **2.49 [1.22,5.09]** |  |
| Student | 0.61 [0.19,1.95] | 0.51 [0.15,1.71] |  |
| Retired | 0.83 [0.21,3.31] | 0.78 [0.19,3.25] |  |
| Unemployed | 1.11 [0.51,2.42] | 1.07 [0.50,2.32] |  |
| Index of multiple deprivation |  |  |  |
| Q1 most deprived | 1 | 1 |  |
| Q2 | 1.01 [0.62,1.64] | 1.02 [0.63,1.65] |  |
| Q3 | 1.06 [0.65,1.75] | 1.06 [0.65,1.75] |  |
| Q4 | 1.39 [0.84,2.29] | 1.40 [0.86,2.31] |  |
| Q5 least deprived | 0.78 [0.44,1.36] | 0.81 [0.47,1.41] |  |
| Unknown | 0.88 [0.52,1.50] | 0.87 [0.51,1.50] |  |
| **Working** | **1.84 [1.19,2.84]** | **1.81 [1.17,2.81]** |  |
| Household size |  |  |  |
| 1 person | 1 | 1 |  |
| 2 persons | 1.18 [0.73,1.92] | 1.18 [0.73,1.92] |  |
| 3+ with children | 1.24 [0.76,2.03] | 1.25 [0.77,2.05] |  |
| 3+ without children | 1.09 [0.63,1.88] | 1.08 [0.62,1.87] |  |
| *Factors associated with Covid* |  |  |  |
| Shielded | 0.75 [0.49,1.15] | 0.78 [0.51,1.18] |  |
| Positive Covid-19 test | 0.90 [0.55,1.46] | 0.91 [0.56,1.45] |  |
| **Times with no access to a medicine** | **1.48 [1.18,1.86]** | **1.50 [1.18,1.89]** |  |
| Times non-prescription medicines were used as substitutes | 1.04 [0.98,1.11] | 1.04 [0.98,1.12] |  |
| **Times immediate supply from pharmacy was required** | **1.38 [1.22,1.56]** | **1.38 [1.22,1.57]** |  |
| Exempt from prescription fee | 1.14 [0.73,1.77] | 1.11 [0.72,1.72] |  |
| Suitable public transport available? | 0.80 [0.53,1.19] | 0.78 [0.52,1.17] |  |
| Help taking medicines at home? | 1.25 [0.80,1.97] | 1.32 [0.85,2.06] |  |
| *Use of digital tools to support medicines management* | | |  |
| Yes, to order or as reminder to take medicines (yes/no) ^c^ | N/A | 1.37 [0.99,1.88] |  |
| Use of digital tools (categorical) |  |  |  |
| No apps or digital tools | 1 | N/A |  |
| **Yes, to remind me to take my medicine** | **1.73 [1.11,2.71]** | **N/A** |  |
| Yes, to help me order my prescriptions | 0.96 [0.64,1.43] |  |  |
| **Yes, to remind me to take and to order my medicine** | **2.37 [1.32,4.28]** | **N/A** |  |
| *Drug/LTC related factors* |  |  |  |
| Number of medicines | 0.91 [0.79,1.04] | 0.91 [0.79,1.04] |  |
| Number of LTCs newly diagnosed since March 2020 | 1.15 [0.93,1.42] | 1.16 [0.94,1.43] |  |
| Number of drugs newly prescribed since March 2020 | 1.07 [0.90,1.28] | 1.07 [0.90,1.28] |  |
| **At least one cardiovascular medicine** | **0.60 [0.38,0.94]** | **0.60 [0.38,0.95]** |  |
| At least one respiratory medicine | 0.63 [0.39,1.00] | 0.63 [0.39,1.00] |  |
| Analgesics for migraine | 1.22 [0.75,2.00] | 1.25 [0.77,2.03] |  |
| Insulin | 0.50 [0.24,1.02] | 0.49 [0.24,1.01] |  |
| **Thyroid and antithyroid medicines** | **0.32 [0.11,0.93]** | **0.32 [0.11,0.93]** |  |
| **At least one medicine used in rheumatic disease and gout** | **0.09 [0.02,0.50]** | **0.10 [0.02,0.48]** |  |
| At least on anxiolytic or hypnotic medicine | 2.02 [0.91,4.48] | 2.13 [0.96,4.70] |  |

^a^ Odds ratio adjusted for age, gender ethnicity, region, socioeconomic group, IMD, work status, household size, new LTCs, number of medicines, exemption from prescription fee, suitable public transport, help taking medicines at home, use of digital tools yes/no (Model 2) and by type (Model1) and , shielding status, positive COVID-19 test, CV-medicine, respiratory medicine, analgesic, insulin, thyroid medicine, medicine for rheumatic disease or gout [significance indicated in bold]; ^b^ 1: Semi or unskilled manual worker, 2: Skilled manual worker, 3: Supervisory or Junior managerial or Professional or Administrator, 4: Intermediate managerial or Professional or Administrative, 5: Higher managerial or Professional or Administrative; ^c^ Use of digital tools reported for question 15 “How did you order your prescription at your GP the last time you renewed your prescription?” and question 26 “Did you use any apps or digital tools to support you to take your medicine?”
